# Conformational switch and multiple supramolecular structures of a newly identified self-assembling protein-mimetic peptide from *Pseudomonas aeruginosa* YeaZ protein

**DOI:** 10.3389/fchem.2022.1038796

**Published:** 2022-12-13

**Authors:** Elettra Fasola, Giulia Alboreggia, Stefano Pieraccini, Francesco Oliva, Fatima Ezzahra Agharbaoui, Michela Bollati, Giovanni Bertoni, Sandro Recchia, Marcello Marelli, Umberto Piarulli, Sara Pellegrino, Silvia Gazzola

**Affiliations:** ^1^ Science and High Technology Department, University of Insubria, Como, Italy; ^2^ Chemistry Department, University of Milan, Milan, Italy; ^3^ CNR and Department of Biosciences, Institute of Biophysics, University of Milan, Milan, Italy; ^4^ Department of Bioscience, University of Milan, Milan, Italy; ^5^ CNR-SCITEC—Istituto di Scienze e Tecnologie Chimiche “Giulio Natta”, Milan, Italy; ^6^ Pharmaceutical Science Department, University of Milan, Milan, Italy

**Keywords:** protein-mimetic peptide, self-assembly, peptide material, motif design, supramolecular gel, YeaZ protein

## Abstract

Protein-mimetic peptides (PMPs) are shorter sequences of self-assembling proteins, that represent remarkable building blocks for the generation of bioinspired functional supramolecular structures with multiple applications. The identification of novel aminoacidic sequences that permit the access to valuable biocompatible materials is an attractive area of research. In this work, *in silico* analysis of the *Pseudomonas aeruginosa* YeaZ protein (*Pa*YeaZ) led to the identification of a tetradecapeptide that represents the shortest sequence responsible for the YeaZ-YeaZ dimer formation. Based on its sequence, an innovative 20-meric peptide, called PMP-2, was designed, synthesized, and characterized in terms of secondary structure and self-assembly properties. PMP-2 conserves a helical character and self-assembles into helical nanofibers in non-polar solvents (DMSO and trifluoroethanol), as well as in dilute (0.5 mM) aqueous solutions. In contrast, at higher concentrations (>2 mM) in water, a conformational transition from α-helix to β-sheet occurs, which is accompanied by the Protein-mimetic peptide aggregation into 2D-sheets and formation supramolecular gel in aqueous environment. Our findings reveal a newly identified Protein-mimetic peptide that could turn as a promising candidate for future material applications.

## 1 Introduction

The supramolecular self-assembly of proteins plays a fundamental role in generating highly complex hierarchical macrostructures with specific biological functions in living organisms (Bai et al., 2016). Protein folding (Kim et al., 2022), the reversible assembly of tubulin (Fletcher and Mullins, 2010) and collagen fibers (Pins et al., 1997), and the interaction of proteins to form complexes with a wide range of bio-functionalities (Bai et al., 2016) are just a few of the numerous examples that nature offers. The discovery that protein-mimetic peptides (PMPs), i.e., shorter sequences of self-assembling proteins, can lead to sophisticated supramolecular structures like nanofibers, micelles, ribbons, or spheres, paved the way to the generation of peptide-based functional bioinspired nanomaterials (Zhang et al., 1993; Goerbitz, 2006; Levin et al., 2020; Locarno et al., 2020; La Manna et al., 2021). These peptide materials are characterized by high biocompatibility and biodegradability, as well as facile synthesis, thus finding several applications in the development of biomaterials for tissue engineering (Gomes et al., 2011; Xu et al., 2015; Berns et al., 2016; Shen et al., 2022), drug delivery (Abbas et al., 2010; Zou et al., 2020), and bioinspired technology (Fu et al., 2009; Assal et al., 2013; Matsuura et al., 2020). In this context, the elucidation of novel protein sequences that permit the access to valuable biocompatible materials is an attractive area of research (Castelletto and Hamly, 2022). Among the identified PMPs (Zhang et al., 2018), sequences deriving from α-helical domains have been less explored due to the intrinsic thermodynamical instability of the helix when it is extracted from the native protein.

On the other hand, α−helices are the most common motif in globular proteins (Mondal and Gazit, 2016), and they are often involved in biological relevant protein-protein interactions (PPIs) (Araghi and Keating., 2016; Zanella et al., 2019). The assembly of two or more α-helices forms coiled-coils, that are a structural element identified in several proteins, and are considered the most stable and regular protein folding known so far (Lupas, 1996; Wolf et al., 1997; Apostolovic et al., 2010; Lupas and Bassler, 2017). For these reasons, self-assembling α−helices provide a promising starting point for the design of novel functional materials with nature-like mechanical features (Lupas and Bassler, 2017).

In this work we present the design of a novel promising PMP, called PMP-2, starting from the recently solved X-ray crystal structure of *Pseudomonas aeruginosa* YeaZ (*Pa*YeaZ, PDB: 4Y0W; Vecchietti et al., 2016). In bacteria, the homodimeric protein YeaZ participates to the formation of protein networks thar are involved in the biosynthesis of an essential tRNA modification, and for this reason YeaZ is receiving growing interest as potential novel target for antibacterial development (Swinehart et al., 2020). Interestingly, the YeaZ homodimers mainly bind through the interaction of the two α2 α-helices to form a coiled-coil-like structure. Based on this evidence, the assembling mode of YeaZ protein was here investigated for the first time through computational studies, and we identified the sequence primarily responsible for the dimer formation within a shorter fragment of the α2 helix. The remarkable self-assembling properties of this new PMP, conveniently functionalized, were studied by a combination of Circular Dichroism Analysis, Electron Microscopy techniques, Dynamic Light Scattering and Infrared Spectroscopy. The results indicated that PMP-2 self-assembles into supramolecular nanofilaments with defined diameter. By modulating the experimental conditions, these nanofibers can transform to 2D-sheets and form gels in aqueous environment, and this transformation is accompanied by a conformational switch of PMP-2 from α-helix to β-sheet secondary structure. Our finding led to the discovery a new PMP deriving from a α-helix domain of a bacterial protein that shows the potential to be used as a building block for generating functional materials (Meng et al., 2014; Mondal and Gazit, 2016; Ruffoni et al., 2016; Li et al., 2017; Zhang et al., 2018). Additionally, the elucidation of the assembly mode of *Pa*YeaZ homodimer that could have potential relevance in drug discovery in the future.

## 2 Materials and methods

### 2.1 Computational studies

The model of YeaZ homodimer was obtained using the published crystal structure (PDB ID code 4Y0W, Vecchietti et al., 2016). We extracted two monomers from the crystal structure. The system was simulated in explicit water solvent with periodic boundary conditions. The protein was described using the amber99SB-ILDN force field (Lindorff-Larsen et al., 2010), while TIP3P model (Jorgensen et al., 1983) was adopted for water. The system was submitted to geometry optimization using the steepest descent method (50,000 steps). Then, we carried out a 200 ps equilibration in the NVT ensemble followed by a second equilibration of further 200 ps in the NPT ensemble. During the equilibration, cα were restrained to crystallographic positions. Equilibration phase was followed by a 100 ns long unrestrained production run in the NPT ensemble. Temperature and pressure were kept constant to their reference values (1 bar, 300 K respectively) through the velocity rescale algorithm (Bussi et al., 2007) and the Berendsen barostat (Berendsen et al., 1984). A 14 Å cutoff was applied for non-bonded interactions and the Particles Mesh Ewald algorithm (Darden et al., 1993) was employed to calculate long range electrostatic interactions. During the MD simulations all bond lengths were constrained to their equilibrium values with the LINCS algorithm (Hess et al., 1997), allowing time step of 2 fs. Simulations and subsequent analysis were performed with the GROMACS 5.0.7 program suite (Van der Spoel et al., 2005). 500 snapshots were extracted from the last 20 ns of the dynamics of the YeaZ complex (one snapshot every 40 ps). The contribution of each of the amino acids at the protein-protein interface to the binding energy has been estimated through computational alanine scanning (CAS). This technique calculates the difference in binding free energy between the protomers forming the complex upon mutation of each of the interfacial residues into alanine (ΔΔG). This evaluation has been performed using the molecular mechanics/Poisson–Boltzmann surface area (MM/PBSA) approach (Massova and Kollman, 2000). A positive ΔΔG value means that the associated residue mutation into alanine reduces the binding energy, while a negative ΔΔG value means that the protein-protein binding energy is increased upon mutation. Residues exhibiting the largest ΔΔG values are thus those contributing most to the complex formation. CAS was performed only for residues located at the interface, that were identified as the ensemble of residues with a different solvent accessible surface area (SASA) in the complex and in the apo-protein. SASA of each residue was calculated using Naccess (S. Hubbard and J. Thornton, 1992–6), ΔG of binding was calculated with the MM/PBSA approach as implemented in the GMXPBSA 2.0 suite (Paissoni et al., 2014). This protocol implicitly assumes that point mutations in the protein do not significantly affect its conformation. The validity of this assumption in computational alanine scanning has been widely confirmed in the literature, when applied to PPIs. A dielectric constant of two was chosen for the protein interior. The initial secondary structures of the synthesized peptides have been obtained with the Pepfold3 server (Lamiable et al., 2016). Each system has been simulated using periodic boundary conditions and explicit solvent model (either water or TFE). The peptides were described using the amber99SB-ILDB (Lindorff-Larsen et al., 2010) force field, while the TIP3P (Jorgensen et al., 1983) model was adopted for water and the Generalized Amber Force Field (GAFF) (Wang et al., 2004) for TFE. Each system was simulated with the protocol described above, except for the production run that was 500 ns long. Three replicas of each simulation have been performed. Cluster analysis has been performed using the Gromos algorithm (Daura et al., 1999) and secondary structure has been calculated with the DSSP software (Kabsch and Sander, 1983).

### 2.2 Peptide synthesis

All the commercially available reagents and resins were used as purchased from Sigma-Aldrich^®^, TCI, Fluorochem^©^, and HPLC grade solvents were employed. Synthesis of peptides was performed by manual solid phase synthesis by the Fmoc-strategy on Fmoc-Gly preloaded Wang resin (commercially reported loading 0.4–0.9 mmol/g). HPLC purifications were performed with SHIMADZU LC-20AP equipped with diode array UV detector and Phenomenex Fusion-RP 80Å column. High resolution mass spectra were obtained with Thermo Fisher Scientific Orbitrap Exploris™ 120 equipped with UHPLC and C18 column. The purities of the synthesized peptides were analyzed by analytical HPLC SHIMADZU LC-20AP equipped with diode array UV detector and C18 column.

### 2.3 Circular Dichroism Analysis

Circular Dichroism experiments were performed on a CD-spectropolarimeter Jasco 820 with a 0.1 cm quartz cuvette. Spectra were acquired from 195 to 250 nm with a 0.1 nm step and 1 s collection time per step, taking three averages. The spectrum of the solvent was subtracted to eliminate interference from cell, solvent, and optical equipment. The CD spectra were plotted as mean residue ellipticity θ (degree·cm^2^·d^−1^·mol^−1^) versus wavelength λ (nm). Noise-reduction was obtained using a Fourier-transform filter program from Jasco. Secondary structure analysis was performed using the CONTIN algorithm (Whitmore and Wallace, 2008) and the reference set 7 (Sreerama and Woody, 2000). CD spectra were registered at 0.07–0.18 mM concentrations, using pure TFE or TFE and buffer solutions to test different pH ranges.

### 2.4 Fourier transform-attenuated total reflection (FT-ATR) analysis

For ATR spectra, Nicolet iS10 Thermo Fisher Scientific spectrometer was employed. Spectra are plotted as Absorbance versus wave numbers (cm^−1^), with resolution 4 cm^−1^ and 32 scans. Solvent subtraction and spectra deconvolution were performed with OMNIC™ Spectra Software.

### 2.5 Trasmission Electron Microscopy analysis

TEM samples were prepared by the same technique from a 2 mM DMSO solution to afford 0.1 mM final concentration in Milli-Q water at different pHs. After deposition onto the carbon/Formvar TEM grid, water was eliminated by lyophilization. Operating conditions: low vacuum mode 1 Torr; accelerating voltage 10 kV; working distance 7.5 mm, GSE detector; magnifications: ×800, ×1,600, ×3,200, ×6,400, and ×12,800. Transmission Electron Microscopy (TEM) and Scanning TEM (STEM) images were collected by a ZEISS LIBRA200FE with in-column Ω-filter and accelerating voltage 200 kV. Samples were treated with UranyLess solution (EMS) for negative staining.

### 2.6 Dynamic Light Scattering analysis

Malvern Zetasizer Nano instrument (Malvern Panalytical, Ltd.) was employed for Dynamic Light Scattering (DLS) analysis, performed at 25°C. The instrument is equipped with a 633 nm solid-state He–Ne laser at a scattering angle of 173°. All the samples used to analyze the stability of the PMP-2 aggregates as a function of different DMSO/water ratio were prepared by solvent displacement technique starting from concentrated solutions in 100% DMSO (0.125, 0.2, 0.5, 2, 4, and 10 mM) to obtain 0.1 mM final concentrations in Milli-Q water, with 80%, 50%, 20%, 10%, 5%, 2.5% and 1% v/v of DMSO, respectively. All the samples used to analyze the stability of the PMP-2 aggregates as a function of concentration were prepared starting from 0.1 mM in Milli-Q water with 5% *v/v* of DMSO and diluting with Milli-Q water to obtain the final concentration of 0.05, 0.01, 0.005, and 0.001 mM. The corresponding refractive index and viscosity data of DMSO/water mixtures are reported in the literature (LeBel and Goring, 1962) and the samples were aged 24 h at room temperature before the measurements.

### 2.7 Scanning Electron Microscopy analysis

Scanning Electron Microscopy (SEM) images were acquired with Philips ESEM FEG Mod. XL 30 without sample coating and in the low vacuum mode (under 1 Torr) of water vapor pressure, to avoid possible morphological modifications. SEM aqueous samples were prepared by solvent displacement technique: DMSO concentrated stock solutions of the synthesized peptides were added dropwise to a proper volume of Milli-Q water at different pHs, to afford a final concentration of 0.5 mM. The samples were lyophilized before the analysis, while the hydrogels were transferred as such.

### 2.8 Thioflavin T fluorescence analysis

Aggregation kinetics were followed using Thioflavin-T (ThT) as fluorescent dye, specific for β sheet-rich structures (LeVine 3rd, 1993). PMP-2, was dissolved in Milli-Q water to reach the final concentration of 0.1 or 2 mM, and heated at 60°C. After 24 h, 100 µl samples were incubated at 25°C in the presence of 20 μM ThT. Fluorescence signal was measured every 20 min for 30 h in a quartz cuvette using a Cary Eclipse Fluorescence Spectrometer. The dye was excited at 450 nm, and the emission was measured at 480 nm.

### 2.9 Hydrogel preparation

To prepare the hydrogels at different concentration, the lyophilized PMP-2 was dissolved in the suitable amount of Milli-Q water (final concentration of the prepared solutions: 0.1, 1, 2, 5, 10, and 20 mM) heating at 60°C for 3 min and sonicating for 1 min. The clear solutions were left cooling at rt. After 24 h of aging the hydrogel formation was verified at concentrations above 2 mM by the stable-to-inversion method. The 20 mM hydrogel was kept at room temperature for 10 months, and it showed complete stability.

## 3 Results and discussion

### 3.1 Design, synthesis and structural characterization of the novel YeaZ-derived PMP

#### 3.1.1 *In silico* identification of the *Pa*YeaZ peptide sequence of the α2 domain responsible for the YeaZ-YeaZ homodimer formation

**FIGURE 1:**
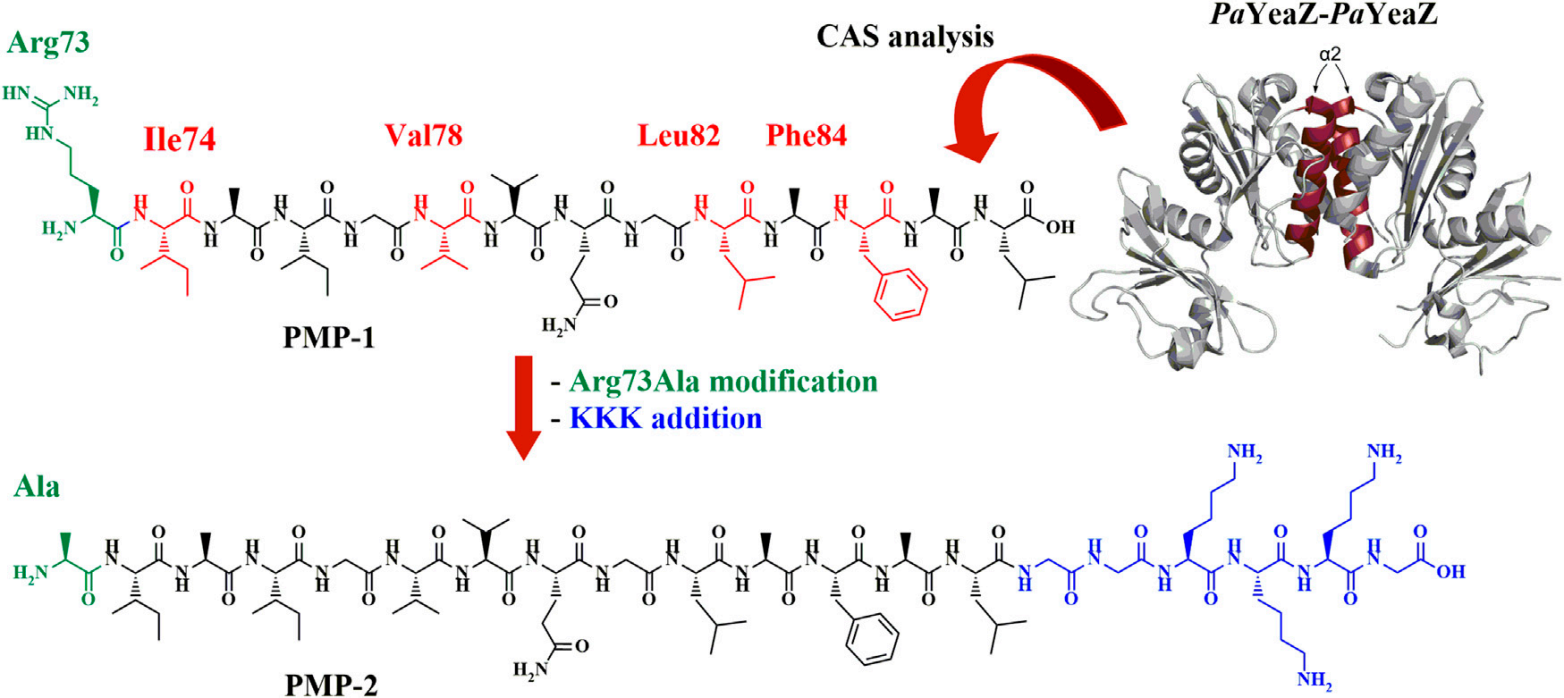
*Pa*YeaZ dimer crystal structure (PDB: 4Y0W, the two α2 helix of each subunit are colored in ruby red), the computationally identified PMP-1 sequence (Arg73-Leu86) and the final amphiphilic PMP-2, object of this study. Hot spots are shown in red in PMP-1, further Arg73Ala modification is highlighted in green and GGKKKG C-terminal sequence is shown in blue.

To predict the sequence responsible for the YeaZ-YeaZ homodimer formation, we performed a computational alanine scanning (CAS), a technique that identifies the smallest subset of amino acids, also known as “hot spots” (Sousa et al., 2007; Keskin et al., 2008; Pieraccini et al., 2009), at the intradimer interface that are responsible for the binding energy of the protein-protein complex formation. The results (Supplementary Material; Section 2.1) revealed four residues having a ΔΔG>2 kcal/mol, namely Ile74, Val78, Leu82, and Phe84, thus crucial for the PPIs. All residues are located on the α2-helix of *Pa*YeaZ, within the coiled-coil-like structure at the protein-protein interface (Supplementary Material; Supplementary Table S1). Based on this prediction, we first selected the sequence RIAIGVVQGLAFAL (Arg73-Leu86) (PMP-1, Figure 1), which was subjected to further modification consisting in the replacement of Arg73 with Ala, as suggested by the better negative ΔΔG value from CAS analysis (Supplementary Material; Supplementary Table S4), and in the addition of a charged tag at the C-terminus. Indeed, the α-helices that naturally assemble in proteins are often amphipathic. Their folding in coiled-coil structure leads to an increased stabilization of the helical structure thanks to additional van der Waals forces, as well as to the shielding of the hydrophobic segments from the aqueous environment (Missirlis et al., 2010; Han et al., 2013). We linked the cationic sequence KKK at the C-terminus of PMP-1 through an additional GG-spacer, generating the amphiphilic structure PMP-2 (Figure 1). The conservation of the helical conformation was ensured by inserting the Lys-tag at the C-terminus, which allows the correct alignment of the charged residues at neutral pH with the helix dipole (Hol, W.G., 1985). In addition, the GG-spacer helps to minimize the effect of the tag to the peptide secondary structure, as reported by Morales and co-workers (Morales and Jimenez, 2019).

Due to the further modification of the sequence, we decided to simulate the secondary structure of the designed PMP-2 by Molecular Dynamics (MD) calculation. MD were carried out both in trifluoroethanol (TFE) and in water to mimic a hydrophobic and hydrophilic environment, respectively. The results suggested that in TFE the α-helical structure is the predominant conformation (28%), and the Lys-tag seems to not hamper the formation of the helix, whereas in water only 3% of helical structure is present, and the major contribution is given by a β-strand structure (15%). Considering that the original sequence is naturally present in the higher hydrophobic core of the protein, it is not surprising that the helix is conserved better in TFE rather than in water. On the other hand, the possible self-assembly process of the PMP showing α-helical predominant conformation, could stabilize the helix also in water thanks to the added polar head, and finally generating supramolecular structures like nanofibers (Lupas and Bassler, 2017). To verify our hypothesis, we synthesized PMP-2 and we studied its secondary structure as well as the predicted self-assembling behavior. Finally, shorter derivatives of PMP-2 were also prepared and investigated to understand the influence of the PMP-1 sequence on the secondary structure and self-assembly properties.

#### 3.1.2 Synthesis of PMP-2 and of the shorter derivatives PMP-3-5

**FIGURE 2:**
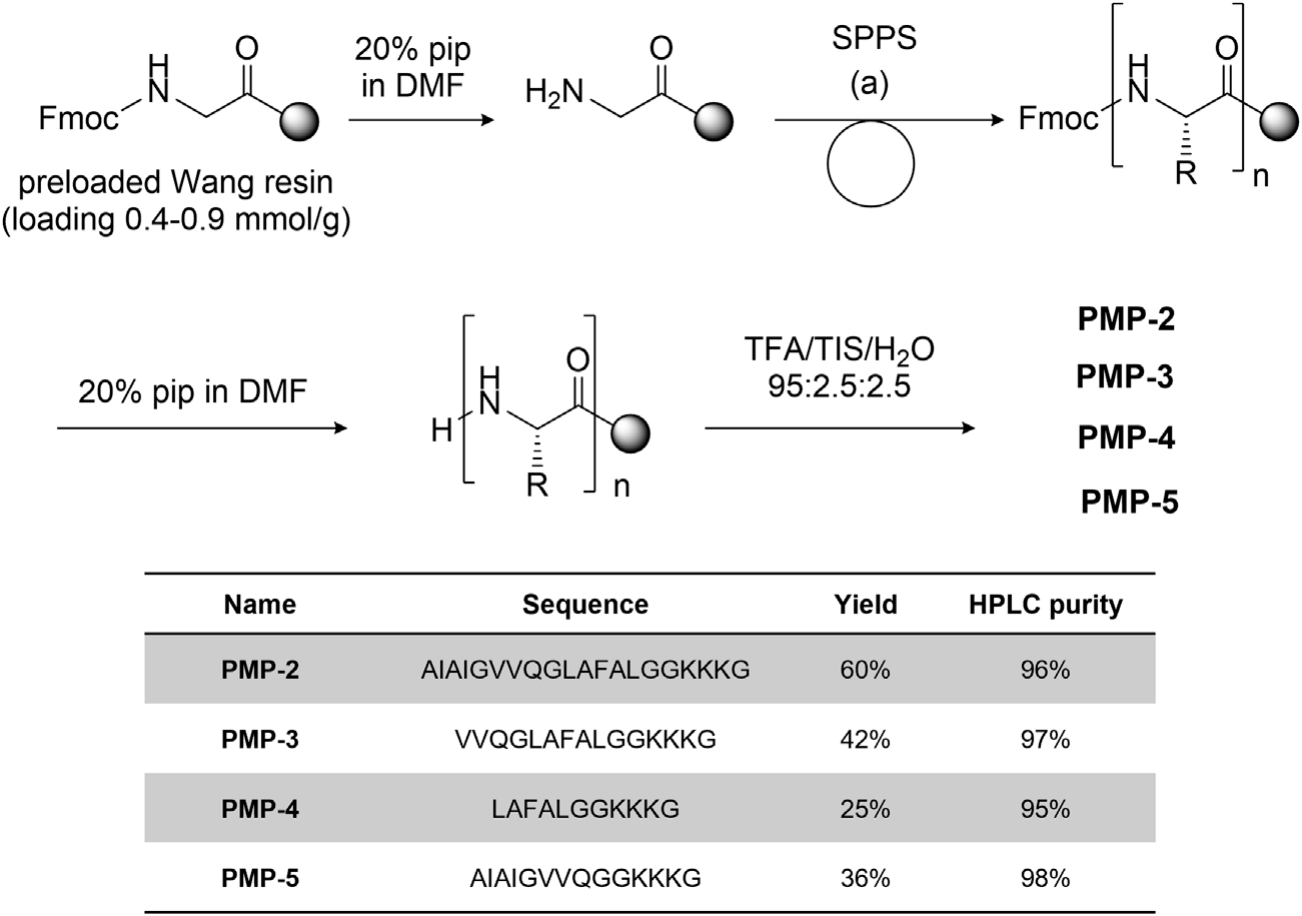
General synthetic scheme [coupling condition (a): conditions: Fmoc-aa-OH 3-5 eq, DIPEA 9-15 eq, COMU 3-5 eq.] and sequence of peptides PMP-3-5 with the isolated yield and HPLC purity.

PMP-2 and its shorter derivatives PMP-3-5 were synthesized by manual solid phase peptide synthesis (SPPS) on Fmoc-Gly preloaded Wang resin (commercially reported loading 0.4–0.9 mmol/g) using the Fmoc strategy. COMU^®^ (3 eq) and *N,N*-diisopropylethylamine (8.5 eq) or *N,N′*-di*iso*propylcarbodiimide (3 eq)/OxymaPure^®^ (3 eq) were used as coupling reagents, depending on the amino acid residue (see Supplementary Information, Section 3.1 for the details). After cleavage from the resin with TFA/H_2_O/TIS 95:2.5:2.5, the peptides was purified by RP-HPLC and fully characterized by HRMS. The general synthetic scheme, yields and purities of the synthesized peptides are reported in Figure 2.

#### 3.1.3 Secondary structure determination by circular dichroism analysis

**FIGURE 3:**
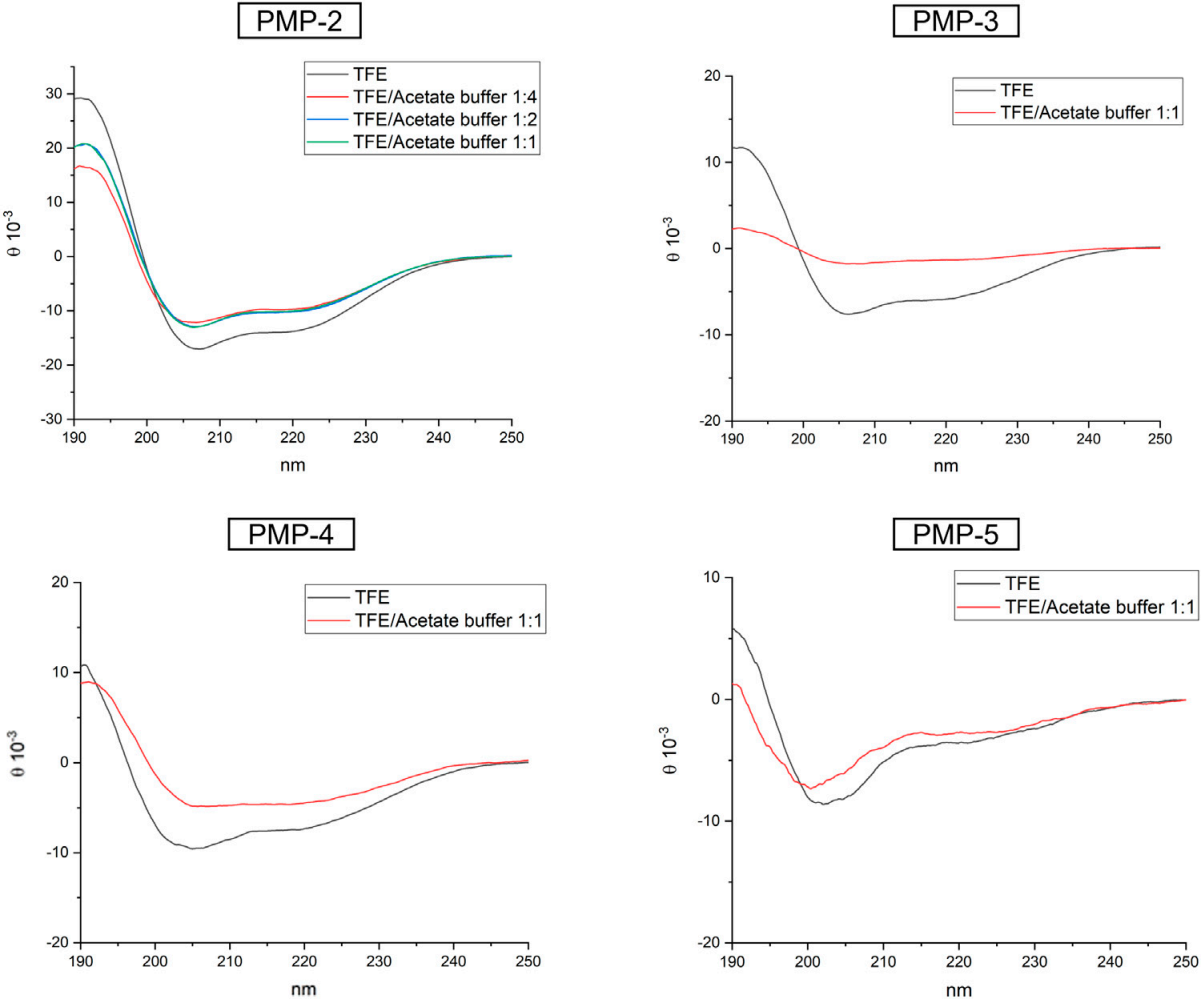
CD spectra of PMP-2-5 measured at 0.1 mM solution in pure TFE (black), or in different ratio of TFE/acetate buffer.

CD experiments were initially performed in TFE at 0.1 mM (Figure 3), to directly compare the MD simulations with the experimental analysis. In TFE, the strong positive band at around 195 nm (amide π-π* transition), the negative band close to 205 nm (amide π-π* transition), and the negative band at 222 nm (amide n-π* transition) observed in the recorded spectra suggested a predominant helical conformation at this concentration, thus confirming the theoretical calculation. CD spectra were also measured at pH = 4 (acetate buffer/TFE 1:1) and pH = 11 (NaOH aqueous solution/TFE 1:1) to modulate the protonation of Lys side chains (Supplementary Material, Supplementary Figure S13) (calculated isoelectric point of PMP-2: 10.4). In both cases the α-helix was conserved, suggesting that the protonation of the C-terminal KKK sequence does not influence the secondary structure of PMP-2. To better understand the influence of the aqueous environment on the stability of the helix, the water content of the solution was increased, and PMP-2 was dissolved in TFE/acetate buffer 1:2 and 1:4 (a major content of water led to precipitation of the peptide). Interestingly, the helical contribution was maintained, indicating a good tendency of the peptide to preserve its α-helix structure even in a more polar solvent (Figure 3).

In order to unravel the molecular determinants of the helical conformation, shorter derivatives of PMP-2 were designed and synthesized (PMP-3-5, Figure 2). PMP-3 and PMP-4 derive from the deletion of subsequent sequences from the N-terminus of PMP-2 (the pentapeptide AIAIG and the non-apeptide AIAIGVVQG respectively), whereas PMP-5 derives from the deletion of the hexapeptide GLAFAL bearing the Phe residue, which represents one of the most important hot-spots for interaction in the *Pa*YeaZ homodimer. A lack of helical structure in all the three derivatives was observed in the MD simulation, and this was confirmed later by CD-spectra (Figure 3). These findings clearly indicated the importance of the whole bacterial α2 sequence of PMP-1 for the maintenance of the α-helix.

### 3.2 Supramolecular aggregation studies of the new PMPs

#### 3.2.1 Trasmission electron microscopy analysis of PMP-2

The remarkable attitude of PMP-2 to self-assemble was analyzed by Transmission Electron Microscopy (TEM). To trigger the self-assembling process of PMP-2 through the polarization of the solvent, the samples were prepared following the solvent displacement technique, which has been used by us (Impresari et al., 2022; Bonetti et al., 2015) and others (Heimburg et al., 1999; Reches and Gazit. 2003; Bucci et al. 2020) with Phe-containing peptides. Lyophilized PMP-2 was dissolved in DMSO to reach a 2 mM concentration, and the resulting solution was added dropwise into Milli-Q water to reach the final concentration of 0.1 mM in 95:5 water/DMSO ratio. Although the CD studies were carried out in TFE, we switched to DMSO due to its higher capability to dissolve PMP-2 compared to TFE, limiting the amount of the hydrophobic solvent in the final aqueous solution. Additionally, the direct comparison of FT-ATR spectra of PMP-2 dissolved in DMSO as well as in TFE (at 10 mM concentration) indicated the adoption of the helical conformation in both cases, thus confirming the possibility of using such solvent for our planned investigations without altering the experimental conditions (Supplementary Material, Supplementary Figures S22, S23).

**FIGURE 4:**
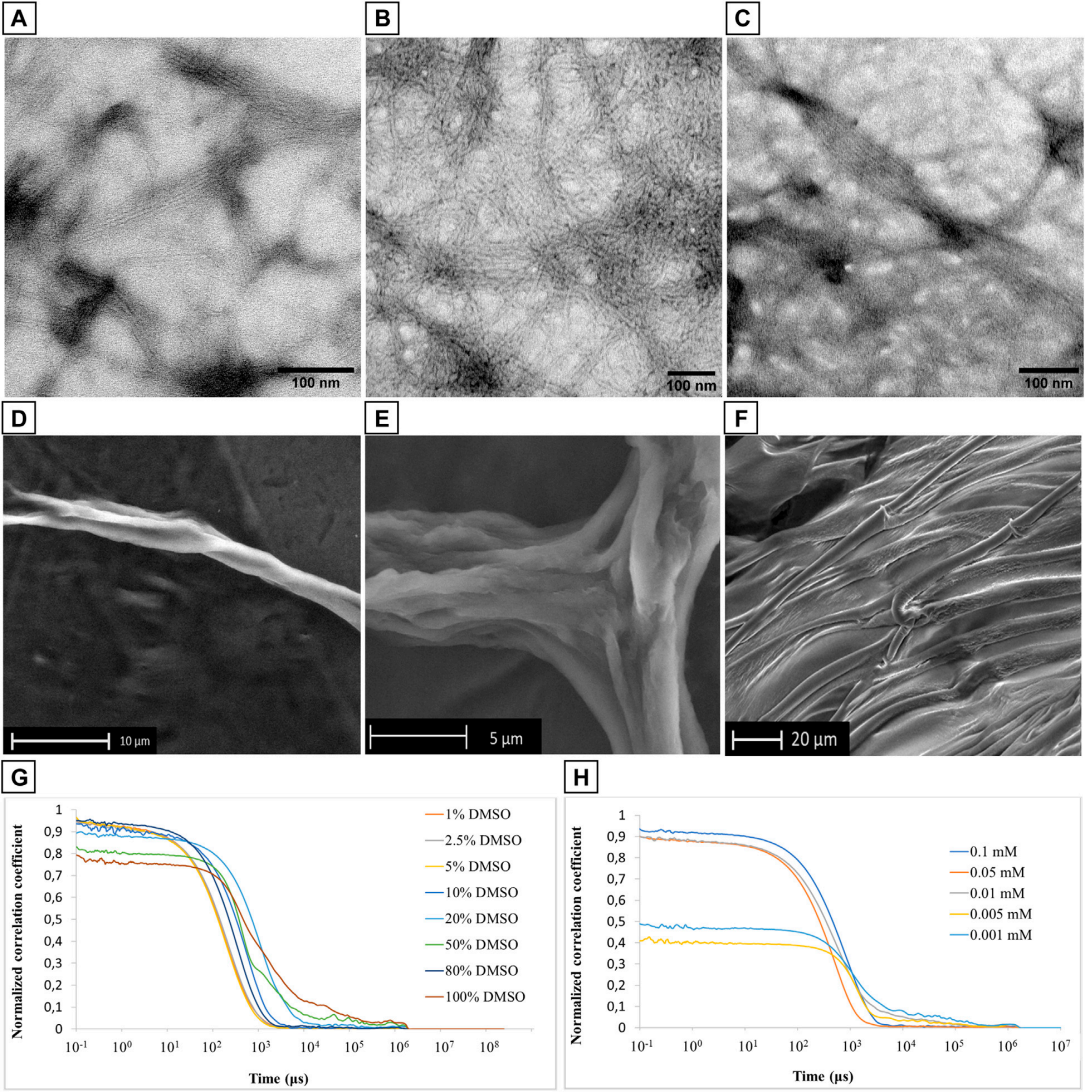
**(A–C)**: TEM micrographs of filamentous assembly of PMP-2 from 0.1 mM solution at pH 7.6 **(A)**, and pH 4 **(B,C)**. **(D–F)**: SEM images of PMP-2 from 0.5 mM solution at measured pH = 7.6. **(G,H)**: DLS measurements of PMP-2: **(G)** Normalized autocorrelation functions at different DMSO/water mixture, and **(H)** normalized autocorrelation functions at different concentration **(H)**.

The resulting 0.1 mM solution was dropped onto a C/Formvar TEM grid, lyophilized, and analyzed. Representative TEM micrographs are reported in Figure 4 (Supplementary Material, Supplementary Figure S18), and they showed the formation of an entangled fibrillar network clearly visible at the 200 nm scale, similar to those fibrillar structures reported in the literature for other α-helical dominant peptide sequences (Potekhin et al., 2001; Cieslik-Boczula 2017). The subunits of these fibers have a diameter of 6 nm, and they are intertwined into bundled filaments (in a range from 3 to 8). Considering the stability of the α-helical conformation of PMP-2 observed through CD analysis by increasing the water content, (Figure 3; Supplementary Material; Supplementary Figure S13), the initial fibrillar formation may effectively arise from the peptide that adopts a helical structure in a hydrophobic environment (DMSO), which is retained in water thanks to the self-aggregation and consequent helical stabilization of PMP-2 (Missirlis et al., 2010; Han et al., 2013). A possible contribution of the positively charged head group to promote the PMP-2 self-assembly into this type of nanostructures is suggested by pH-dependent aggregation studies. Whereas at pH = 4 a similar behavior to the neutral aqueous solution was observed with the formation of the same filaments (Figures 4B,C), at pH 11 the observed few filaments co-existed with an undefined reticular structure, which is not stable under the experimental conditions. (Supplementary Material; Supplementary Figure S18D). Although this result is still under investigation, the possible control of the self-assembly by changing the pH might be an advantage in terms of future application as functional material.

#### 3.2.2 Scanning Electron Microscopy analysis of PMP-2

To further investigate the capacity of these fibers to bundle into bigger filaments, Scanning Electron Microscopy (SEM) analyses were carried out at an increased concentration of 0.5 mM in water obtained by the solvent displacement technique. SEM images were collected in the low vacuum mode, under 1 Torr of water vapor pressure, to avoid possible morphological modifications induced by a preliminary gold coating. As shown in Figures 4D,E, PMP-2 forms helical fibers of several micrometers size, resulting from the aggregation of multiple bundled filaments. These micrometer long fibers are well visible at the edge of the sample holder, while in the middle we found the co-existence of parallel fibers and a 2D sheet-like structure as the one shown in Figure 4F. This effect might be a consequence of a concentration gradient in the sample drop during the lyophilization (increasing from the edge to the center), thereby a conformational switch, and consequently a morphological transformation, can occur as function of a different concentration. To support this hypothesis, we performed FT-ATR and ThT fluorescence analysis, which are reported in paragraph 2.3.

#### 3.2.3 Dynamic Light Scattering analysis

Dynamic Light Scattering analysis was used to analyze the stability of the PMP-2 aggregates as a function of different DMSO/water ratio at 0.1 mM. A very poor scattering power was observed when PMP-2 was dissolved in 100, 80, and 50% *v/v* DMSO/water, whereas a more stable correlogram was obtained with 20% and 10% of DMSO, indicating the formation of some stable aggregates. A perfect overlap of the correlograms was achieved when the water content was increased, and PMP-2 was dissolved in 5, 2.5, and 1% *v/v* DMSO/water solutions (Figure 4G). The obtained curves, reproducible over several acquisitions, confirmed the presence of stable aggregates at the conditions used for TEM and SEM analysis (5% and 2.5% DMSO *v/v* respectively). The aggregation tendency of PMP-2 was also investigated as function of concentration. The 0.1 mM solution of PMP-2 dissolved in 2.5% v/v DMSO/water was diluted to 0.05 and 0.001 mM with Milli-Q water, and a good fitting was obtained in both cases (Figure 4H). By contrast, no reliable correlograms were obtained at 0.005 and 0.0001 mM, probably due to the poor scattering power of the solutions and to the limit of detection of the instrument.

#### 3.2.4 Electron microscopy analysis of PMP-3-5

TEM and SEM studies were carried out for the shorter derivatives PMP-3-5 by preparing the respective final 0.1 mM solutions and 0.5 mM solutions by solvent displacement. The obtained micrographs showed a different behavior of the three peptides compared to PMP-2, and no similar nanofibers were observed (Supplementary Material, Supplementary Section 4.1). These results highlighted the importance of the α-helix conformation assumed by the entire bacterial amino acid sequence present in PMP-2 to generate nanofibers.

### 3.3 Conformational switch

#### 3.3.1 FT-ATR studies

**FIGURE 5:**
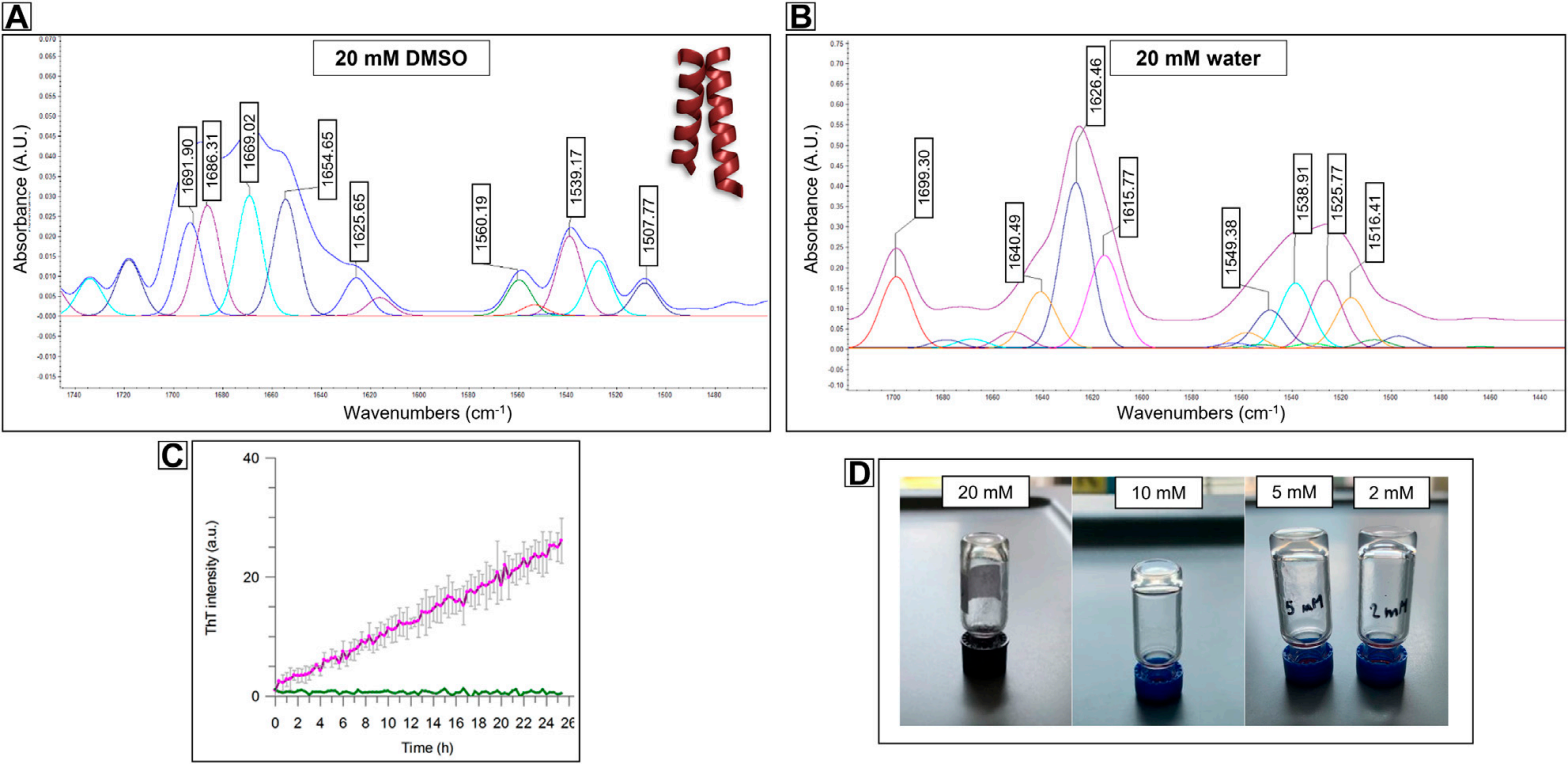
FT-ATR spectra of Compound PMP-2 at 20 mM concentration in **(A)** DMSO and **(B)** in water. In DMSO the pentameric signal of a coiled-coil like structure can be recognize, while in water the β-sheet conformation is predominant. **(C)** Stable-to-inversion gel test on PMP-2 in Milli-Q water at 20 mM, 10 mM and 5 mM and 2 mM, respectively. **(D)** ThT fluorescence assays showing 2 mM PMP-2 (purple trace) aggregation over 24 h. The concentration of the control PMP-2 is 0.1 mM (green trace).

The experimental evidence observed during the SEM analysis of PMP-2, in particular the co-existence of parallel fibers and a 2D sheet-like structure showed in Figure 4F, suggested that PMP-2 can adopt different conformations by varying the concentration. In addition, the increasing contribution of β-sheet conformation observed in the CD spectra upon changing the hydrophilicity of the solution (from 10% in pure TFE to 17% in the 1:2 and 1:4 TFE/water solutions), supported the hypothesis that also the hydrophilicity could have a crucial role in the final secondary structure of PMP-2. A change in the solution parameters can thus trigger a conformational switch, and this can lead to different aggregates. This tunable behavior of specific sequences is recently gaining growing attention because of the possibility to generate multiple morphologies from a single compound (Kaur at al., 2020; Ghosh et al., 2022). The conformation of PMP-2 in the highly concentrated 20 mM DMSO solution was thus investigated by AFT-ATR analysis, where a dimeric coiled-coil profile deriving from helical peptide self-assembly could be recognized (Figure 5A) by the pentameric signals revealed by the deconvolution process (Kammerer et al., 2004; Hendricks et al., 2017). This behavior is indicative of the attitude of the bacterial sequence to self-assemble as coiled coil when located in the hydrophobic environment of the native *Pa*YeaZ protein. As showed by the spectra obtained from the 10 mM DMSO solution, this coiled coil-like structure is destabilized by dilution, although still present, suggesting that molecular interactions are needed to stabilize the helical conformation.

For a direct comparison, we tried to achieve a similar concentration of PMP-2 in water although its low solubility at room temperature. Interestingly, after sonicating and heating at 60°C, the 20 mM solution of PMP-2 in Milli-Q water, a transparent hydrogel-like morphology was obtained after aging overnight. The Minimum Gelation Concentration (MGC) was found at 2 mM (3.8 mg/ml) by the stable-to-inversion test method, and the final pH of the hydrogel was 8.4 (Figure 5D). In contrast to the DMSO solution, the FT-ATR spectrum of the 20 mM hydrogel clearly shows a β-sheet conformation, with a peak at 1,626 cm^−1^, and a peak at 1,690 cm^−1^ that can be attributed to a β-strand aggregation (Figure 5B). Same FT-ATR spectra profiles were obtained with the more diluted hydrogels at concentration 10, 5, 2 mM, suggesting the occurring of a conformational switch from α-helix to β-sheet, as we previously hypothesized. (Supplementary Material, Supplementary Figure S25).

#### 3.3.2 ThT fluorescence analysis

To assess the secondary structure content of the hydrogels, we tested the propensity of PMP-2 dissolved in Milli-Q and heated at 60°C to form β-rich aggregates *in vitro* by Thioflavin T (ThT) assay. This benzothiazole salt is a commonly used fluorogenic compound specific for β-amyloid-like structures. Upon the binding to β-sheet-rich structures, the dye displays enhanced fluorescence at 480 nm. Figure 5C shows the increased ThT signal of PMP-2 at the lowest hydrogel concentration of 2 mM over 2 days’ incubation at 25°C, suggesting the formation of β-like structure, while at 0.1 mM concentration, at which PMP-2 does not form the gel, no signal increase was observed.

#### 3.3.3 Electron microscopy analysis

**FIGURE 6:**
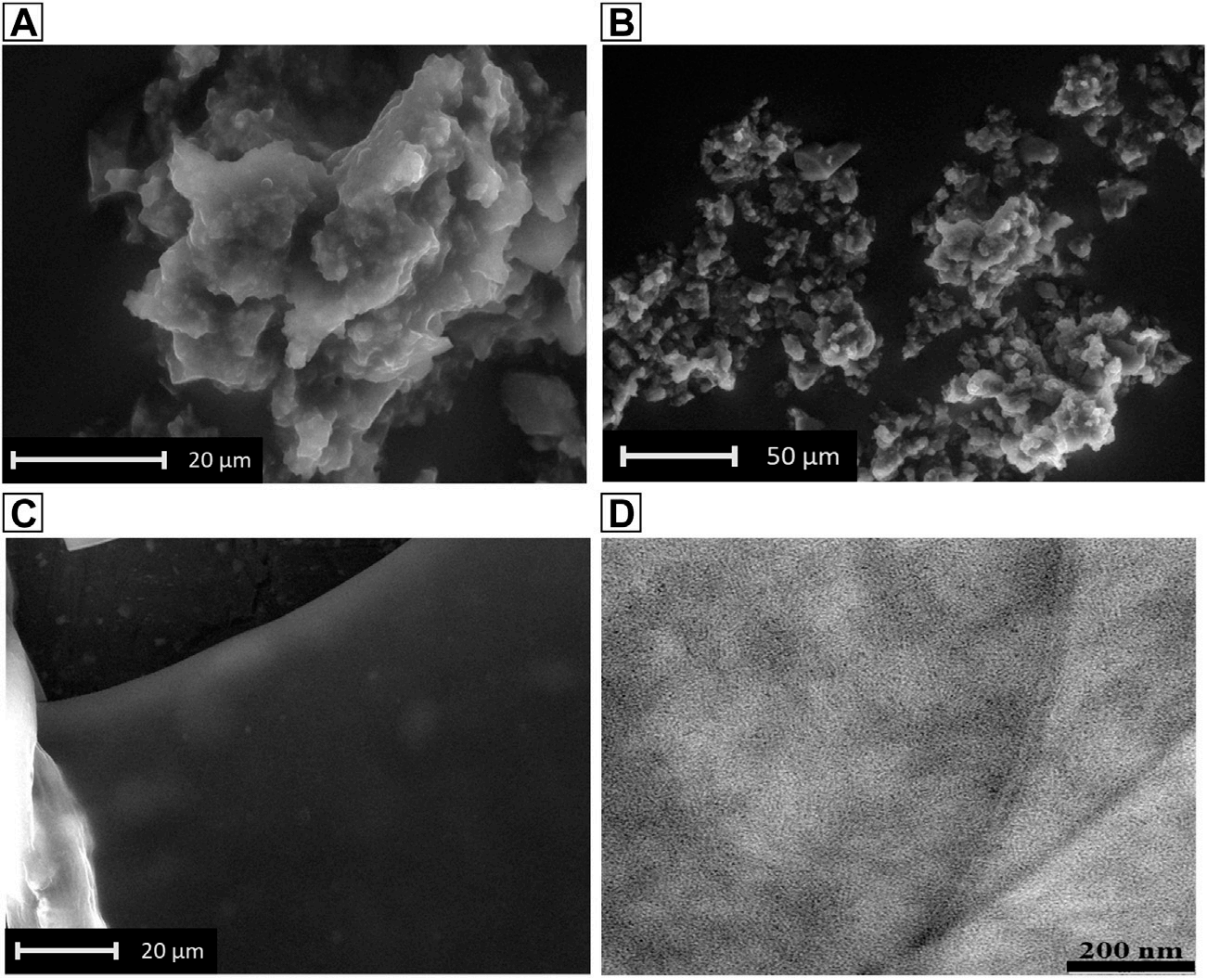
SEM images of the hydrogel in dry form **(A)**, and in wet form **(B,C)**. **(D)**: TEM image of the 2 mM hydrogel in dry form.

The effect of the conformational switch was observed through Electron Microscopy Techniques. In particular, the 20 mM hydrogel was analyzed by SEM as wet form and dried form. In the first case, SEM analysis showed a fractal-like morphology that alternatively grows and gets smaller through the successive wet and dry cycles directly into the instrument, (Figures 6A,B). This experiment was done by changing the relative humidity directly inside the environmental SEM chamber, through slight changes in the cooled sample holder, and suggests the ability of the hydrogel to regenerate by simply absorbing aqueous vapors. By contrast, the thick film observed analyzing the xerogel (Figure 6C), support the correlation between the β-sheet secondary structure and 2D-sheet formation previously observed in Figure 4E. The fine structure of the film was subsequently observed by TEM (from the 2 mM concentration) that showed again the formation of a thick 2D-sheet, in which the typical hydrogel fiber network can be distinguished (Figure 6D).

## 4 Conclusion

In this work, we reported the discovery of a novel PMP sequence deriving from the α-helix domain α2 of *Pa*YeaZ that undergoes a conformational switch from α-helix to β-sheet, and it is able to generate multiple morphologies. This essential protein in bacteria forms protein networks to exploit its biological function, and the X-ray structure of the homodimer was recently solved (Vecchietti et al., 2016). By molecular dynamic simulations and CAS analysis, we identified the shortest amino-acidic sequence (PMP-1) responsible for the dimer formation. A further chemical modification was introduced to stabilize the helical conformation and increase the water solubility of the otherwise very hydrophobic PMP sequence. The remarkable attitude of the helical PMP-1 to self-assemble was investigated after chemical modifications that led to the final peptide structure PMP-2. Overall, conformational analysis by CD and FT-ATR experiments indicate that PMP-2 conserves its helical character in hydrophobic environment like TFE and DMSO. At the highest reachable concentration in DMSO (20 mM), a dimeric coiled-coil profile can be recognized by FT-ATR spectra. Interestingly, the pose of the interacting helices of the *Pa*YeaZ homodimer in the X-ray crystal structure can be attributed to a dimeric coiled-coil as well, thus giving a further indication that the helical conformation of *Pa*YeaZ in solution (at least in hydrophobic solvents or in diluted water solutions, <0.5 mM) is maintained in the solid state (Vecchietti et al., 2016). Additionally, an increased water content up to 80% does not abolish the α-helix as shown by CD analysis, proving a stabilization of this secondary motif also in water, although to a lower extent. The nanoaggregates of PMP-2 formed in 95:5 water/DMSO solution at 0.1 mM concentration were studied by DLS and TEM, the latter showing the formation of filaments that are formed by the intertwining of 3–8 nanofibers having diameter of 6 nm. By increasing the concentration and the water content (97.5:2.5 water/DMSO at 0.5 mM), a conformational transition occurs, and PMP-2 adopts a β-sheet structure, as shown by the FT-ATR spectra. Probably, higher concentrations in a polar solvent emphasize the amphiphilic character of PMP-2, thus facilitating β-sheet rich structures as reported in the literature (Kammerer et al., 2004; Hendricks et al., 2017). Above 2 mM, the formation of a hydrogel-like morphology is recognized by dissolving **PMP-2** in 100% Milli-Q water and heating the sample to 60 °C. The secondary β-sheet structure adopted by the **PMP-2** also in this morphology was proven by FT-ATR and ThT analysis, confirming the occurrence of a conformational transition.

The unraveling of the molecular features (sequences, secondary structures, and intermolecular forces) that determine the proteins folding and assembly, is the driving force for the design of supramolecular architectures with potential useful applications (Baldwin, 1989). The additional ability of certain protein sequences to switch to different secondary structural elements (α-helix, β-sheet), as well as to form different aggregate types by a fine tuning of the external conditions, is known, and represents a topic of high interest due to the possibility to generate multi-stimuli responsive materials from a single building block. In addition, naturally occurring conformational switches are often involved in important biological processes (Wang et al., 2019). Thus, our findings may have potential utility for generating novel responsive biocompatible materials, as well as have relevance to fostering the understanding of the bacterial protein network formation, which could turn into a new biological target. Further investigations currently ongoing in our laboratories aim at determining the antimicrobial activity and cytotoxicity of PMP-2, and the mechanical and functional properties of this hydrogel like material.

## Data Availability

The original contributions presented in the study are included in the article/Supplementary Material, further inquiries can be directed to the corresponding authors.
