# Supplementary material for: Conformational switch and multiple supramolecular structures of a newly identified self-assembling protein-mimetic peptide from Pseudomonas aeruginosa YeaZ protein
Source: Front Chem. 2022 Dec 13;10:1038796. doi: 10.3389/fchem.2022.1038796 (PMC9792601; doi:10.3389/fchem.2022.1038796)
Supplement: Supplementary file 1 [file DataSheet1.PDF]

Supplementary Material

Table of contents

1. **Table of peptide solutions' concentrations**
2. **Computational studies**
   2.1 Peptide Design: Computational Alanine Scanning
   2.2 Molecular Dynamic Simulations of Peptides
3. **Synthesis and structural characterization**
   3.1 Solid Phase Peptide Synthesis of Compounds **PMP-2-5**: general procedure
   3.2 HPLC traces of Compounds **PMP-2-5**
   3.3 TIC (ESI+) and HRMS spectra of Compounds **PMP-2-5**
   3.4 Circular dichroism spectra
4. **Aggregation analysis**
   4.1 SEM and TEM images
   4.2 FT-ATR spectra
5. **Preparation of the PMP-2-based hydrogels**
6. **References**

1 Tables of peptide solutions' concentrations

DMSO solutions	mM	mg/mL
**PMP-2**	20	38
**PMP-3**	34	50
**PMP-4**	46	50
**PMP-5**	38	50

Supplementary Table S1. DMSO stock solutions before solvent displacement.

PMP-2 hydrogels		
mM	mg/mL	%*w/w*
20	38	3.81
10	19	1.90
5	9.5	0.95
2	3.8	0.38
1	1.9	0.19
0.1	0.2	0.02

Supplementary Table S2. PMP-2 hydrogels concentrations with different units.

2 Computational studies

2.1 Peptide design: Computational Alanine Scanning

First Subunit		Second Subunit	
Residue	ΔSASA (nm^2)	Residue	ΔSASA (nm^2)
Ala 68	0.000	Ala 68	0.000
Phe 69	0.006	Phe 69	0.079
Thr 70	**0.200**	Thr 70	**0.335**
Gly 71	0.059	Gly 71	0.000
Val 72	0.003	Val 72	0.000
Arg 73	**1.000**	Arg 73	**0.793**
Ile 74	**0.932**	Ile 74	**0.627**
Ala 75	0.000	Ala 75	0.000
Ile 76	0.030	Ile 76	**0.100**
Gly 77	**0.379**	Gly 77	**0.428**
Val 78	**0.299**	Val 78	**0.295**
Val 79	0.000	Val 79	0.000
Gln 80	**0.537**	Gln 80	**0.588**
Gly 81	**0.425**	Gly 81	**0.432**
Leu 82	**0.276**	Leu 82	**0.278**
Ala 83	0.000	Ala 83	0.000
Phe 84	**1.175**	Phe 84	**1.251**
Ala 85	**0.671**	Ala 85	**0.654**
Leu 86	**0.111**	Leu 86	**0.111**
Gln 87	0.097	Gln 87	**0.219**
Arg 88	0.000	Arg 88	0.000

Supplementary Table S3. SASA difference of residues in apo-protein and in the complex. Non-zero ΔSASA is associated to residues located at the protein-protein interface. In both subunits the interface is largely made by residues belonging to the α2 helix.

First Subunit		Second Subunit	
Mutation	ΔΔG ($kcal{\cdot}mol^{-1}$)	Mutation	ΔΔG ($kcal{\cdot}mol^{-1}$)
Thr70Ala	-0.5497	Thr70Ala	0.3585
Val72Ala	0.0956	Val72Ala	0.0717
Arg73Ala	-13.0972	Arg73Ala	-8.6279
Ile74Ala	**3.5133**	Ile74Ala	0.6214
Ile76Ala	0.2629	Ile76Ala	0.8843
Val78Ala	**2.1032**	Val78Ala	1.5774
Gln80Ala	-0.1673	Gln80Ala	-1.4340
Leu82Ala	**3.2982**	Leu82Ala	**2.2705**
Phe84Ala	**7.5524**	Phe84Ala	**10.6594**
Leu86Ala	1.6491	Leu86Ala	1.3862
-	-	Gln87Ala	-0.9560

Supplementary Table S4. CAS results for the two *Pa*YeaZ subunits forming the dimer. The "hot spots" residues belong to the α2 helix.

2.2 Molecular Dynamics Simulations of Peptides

Secondary structure composition of the peptides in Water and TFE solution (calculated from MD data):

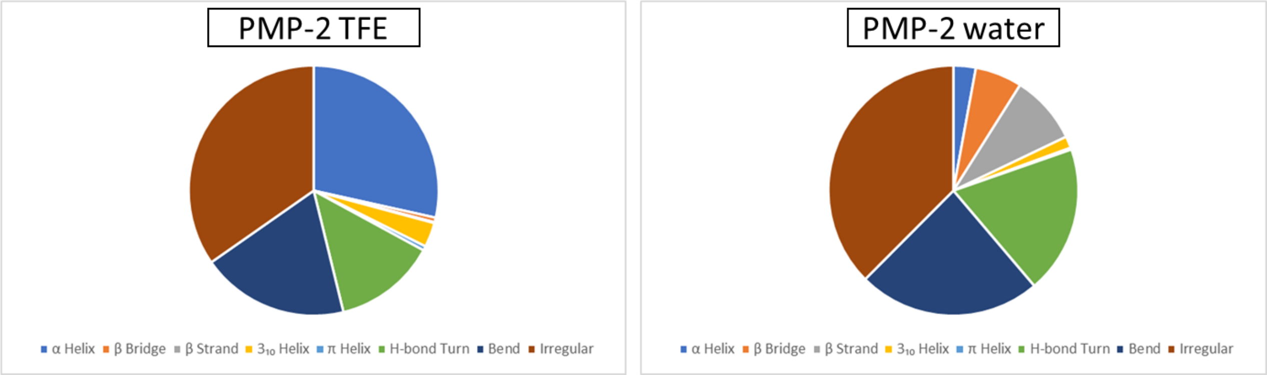

Supplementary Figure S1. Calculated secondary structure composition of **PMP-2** in TFE and water.

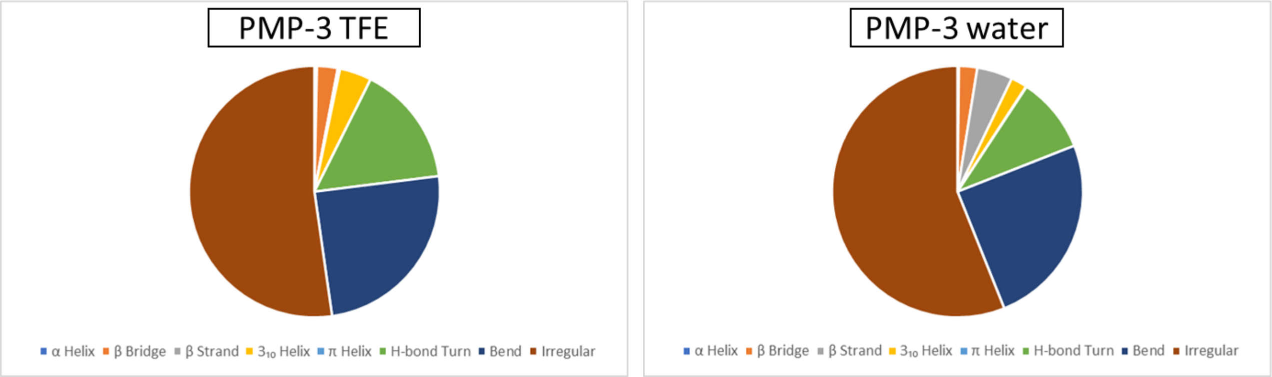

Supplementary Figure S2. Calculated secondary structure composition of **PMP-3** in TFE and water.

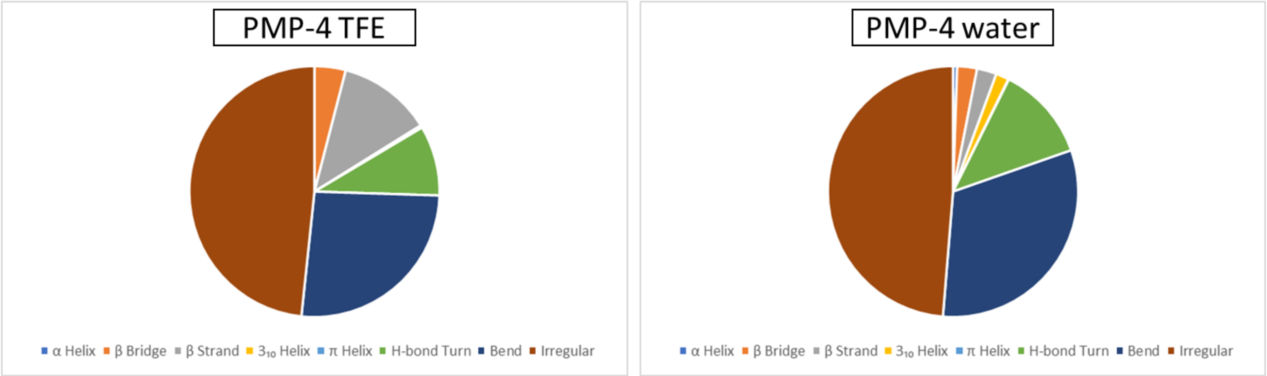

Supplementary Figure S3. Calculated secondary structure composition of **PMP-4** in TFE and water.

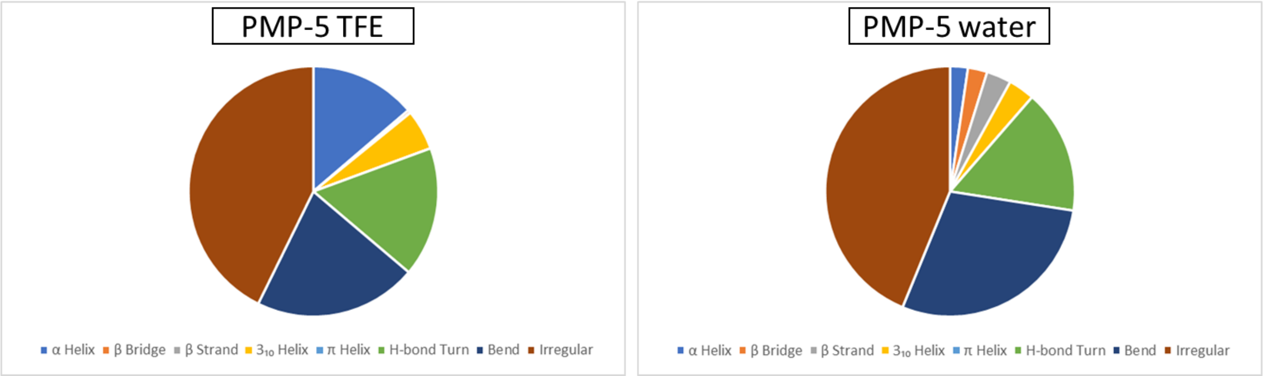

Supplementary Figure S4. Calculated secondary structure composition of P**MP-5** in TFE and water.

3 Synthesis and structural characterization

3.1 Solid Phase Peptide Synthesis of Compounds PMP-2-5: general procedure

As reported in **Scheme S1**, all the peptides were synthesized using manual Solid Phase Peptide Synthesis Fmoc-strategy at 0.1 mmol scale. Fmoc-Gly Wang resin (1 eq, commercially indicated loading: 0.4-0.9 mmol/g) was used as solid support.

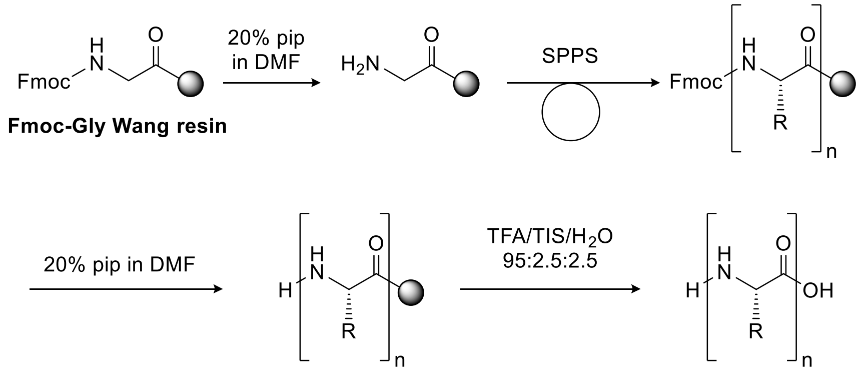

Supplementary Scheme S1. General SPPS procedure.

The resin was initially swollen for 30 minutes in DMF at rt. Following steps are repeated for each amino acid (3 eq):

i) Fmoc-protecting group was removed from the N-terminus by treatment of the resin with 20% *v/v* piperidine in DMF (3 x 3 minutes);

ii) The resin was washed alternating DMF and IPA (4 cycles, 1 minute for each solvent, 1 mL/0.1 mmol of resin);

iii) The amino acids were dissolved in DMF and deprotonated by DIPEA (8.5 eq) under stirring for 3 minutes; COMU (3 eq) was employed as activating agent: after 3 minutes under stirring, the orange to intense red solution (depending on the amino acid) was added to the syringe containing the growing peptide on the solid support, and it was left reacting for 1 hour.

iv) The remaining solvent was discarded, and the resin was washed alternating DMF and IPA (4 cycles, 1 minute for each solvent, 1 mL/0.1 mmol of resin).

Once the desired peptide sequence was obtained, the cleavage from the resin and the concomitant deprotection of the side chains (Boc on Lys, Trt on Gln) were carried out by treatment of the peptide-bound resin with a TFA/water/TIS solution (95:2.5:2.5 *v/v/v*, 2 mL/0.1 mmol of resin). The mixture was stirred at rt for 90 minutes. The resin was washed with TFA and then filtered off. The crude product was precipitated by ice-cold Et_2O (3 x 15 mL) and finally, suspended in DCM and evaporated *in vacuo*.

The peptides were solubilized in CH_3CN/H_2O 1:1 at concentrations up to 20 mg/mL and purified by preparative RP-HPLC SHIMADZU LC-20AP equipped with diode array UV detector and Phenomenex Fusion-RP 80Å.

3.2 HPLC traces of Compounds PMP-2-5

Peptides were analyzed by analytical RP-HPLC SHIMADZU LC-20AP equipped with diode array UV detector and C-18 column.

Flux: 1 mL/min
UV wavelength: 200-203 nm
Solvent A: H_2O milliQ, 0.1% HCOOH
Solvent B: CH_3CN

Gradient 1 (PMP-2, 4, 5): isocratic 98%A–2%B for 5 minutes; from 98%A–2%B to 75%A–25%B in 5 minutes; from 75%A–25%B to 30%A–70%B in 5 minutes; isocratic 30%A–70%B for 10 minutes.

Gradient 2 (PMP-3): isocratic 98%A–2%B for 5 minutes; from 98%A–2%B to 75%A–25%B in 5 minutes; from 75%A–25%B to 30%A–70%B in 5 minutes; isocratic 30%A–70%B for 3 minutes; from 30%A–70%B to 98%A–2%B in 7 minutes.

- **Compound PMP-2**

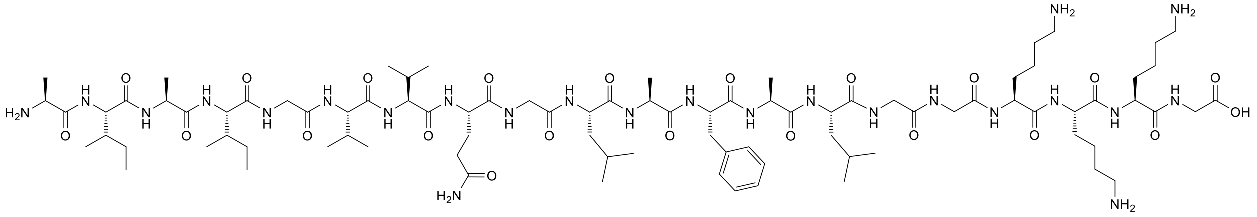

Isolated yield: 60%

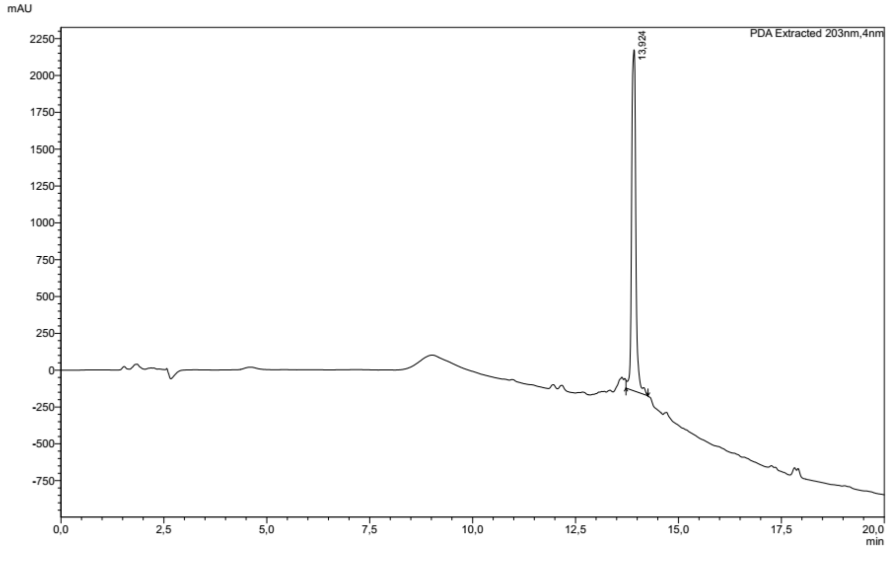

Purity: 96%

Supplementary Figure S5. HPLC trace of **PMP-2**, dissolved in CH_3CN/H_2O 1:1.

- **Compound PMP-3**

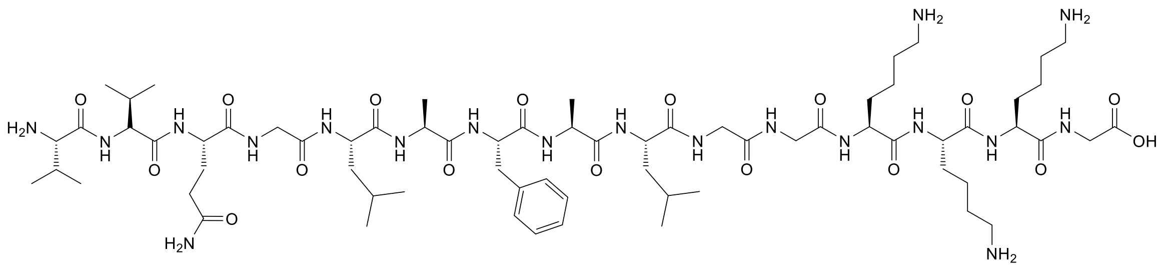

Isolated yield: 42%

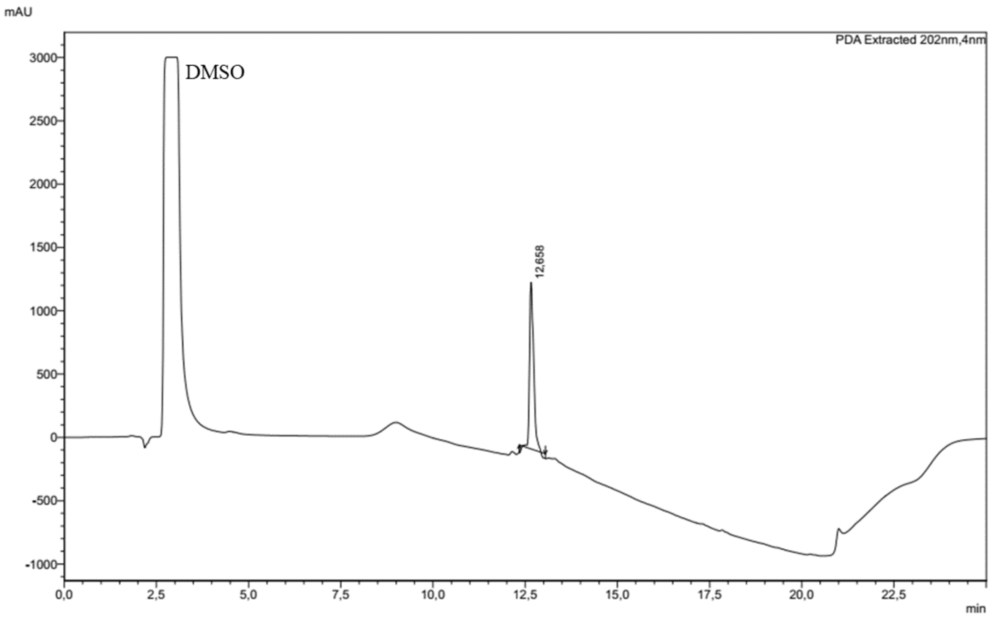

Purity: 97%

Supplementary Figure S6. HPLC trace of **PMP-3**, dissolved in CH_3CN/H_2O 1:1 and one drop of DMSO.

- **Compound PMP-4**

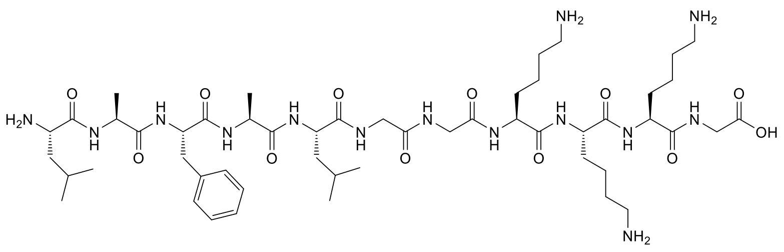

Isolated yield: 25%

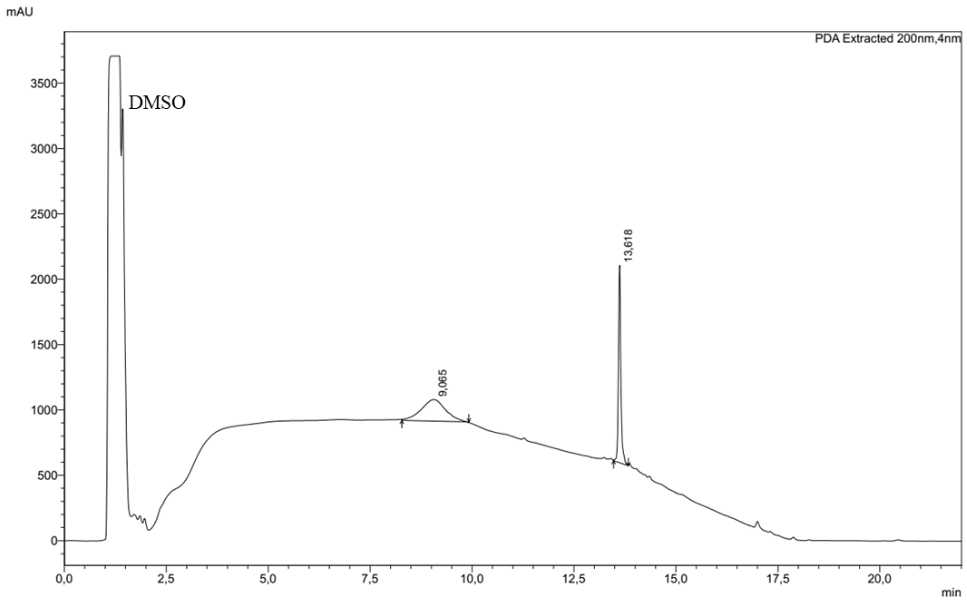

Purity: 95%

Supplementary Figure S7. HPLC trace of **PMP-4**, dissolved in CH_3CN/H_2O 1:1 and one drop of DMSO.

- **Compound PMP-5**

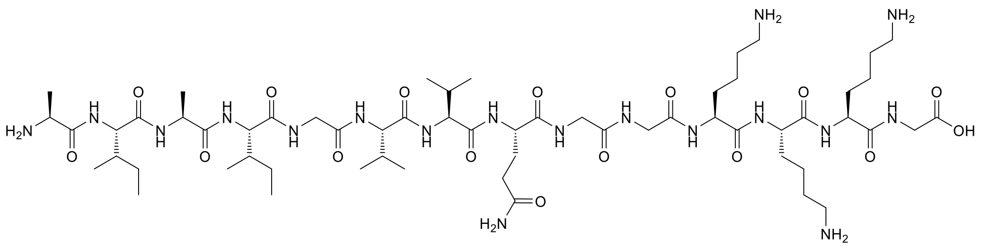

Isolated yield: 36%

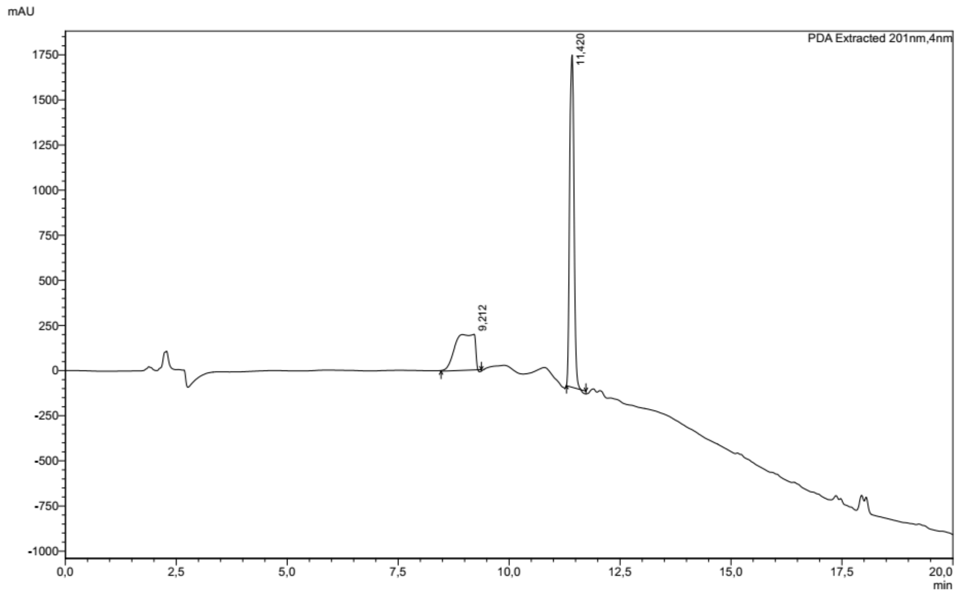

Purity: 98%

Supplementary Figure S8. HPLC trace of **PMP-5.**

3.3 TIC (ESI+) and HRMS spectra of Compounds PMP-2-5

- **Compound PMP-2**

Chemical Formula: $C_{88}H_{152}N_{24}O_{22}$

MW = 1898.33 g/mol

m/z [+2]: calculated = 950.0912 found = 950.0824

m/z [+3]: calculated = 633.3910 found = 633.3898

m/z [+4]: calculated = 475.5451 found = 475.5448

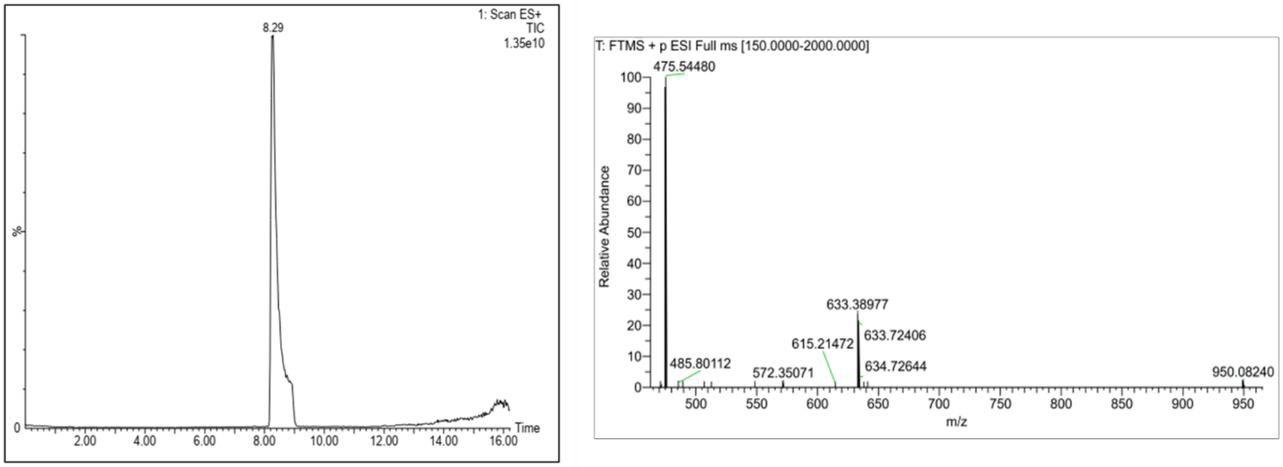

Supplementary Figure S9. TIC and HRMS spectrum of **PMP-2.**

- **Compound PMP-3**

Chemical Formula: $C_{68}H_{117}N_{19}O_{17}$

MW = 1472.80 g/mol

m/z [+2]: calculated = 736.9510 found = 736.9484

m/z [+3]: calculated = 491.6364 found = 491.6349

m/z [+4]: calculated = 368.9792 found = 368.9781

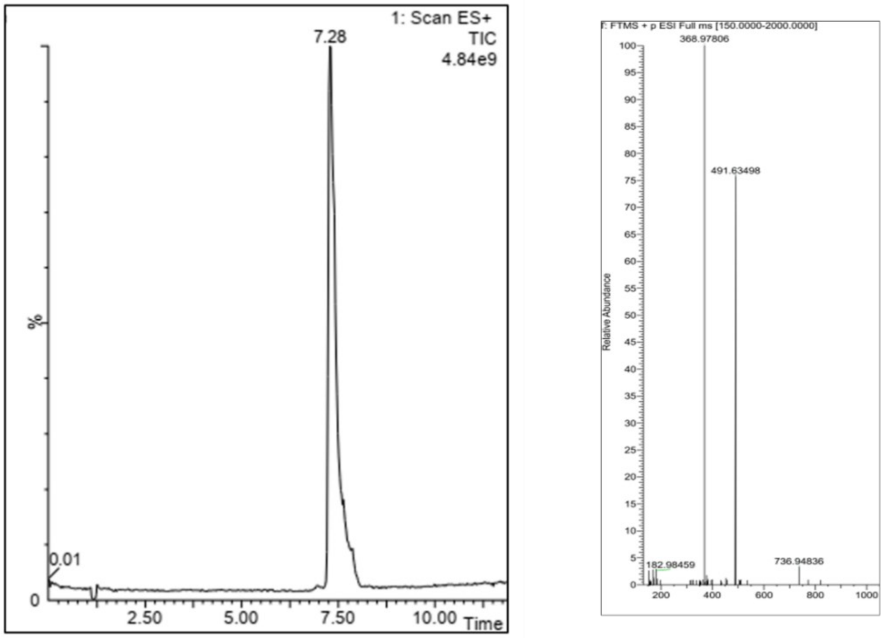

Supplementary Figure S10. TIC and HRMS spectrum of **PMP-3.**

- **Compound PMP-4**

Chemical Formula: $C_{51}H_{88}N_{14}O_{12}$

MW = 1089.35 g/mol

m/z [+2]: calculated = 545.3426 found = 545.3411

m/z [+3]: calculated = 363.8975 found = 363.8967

m/z [+4]: calculated = 273.1749 found = 273.1744

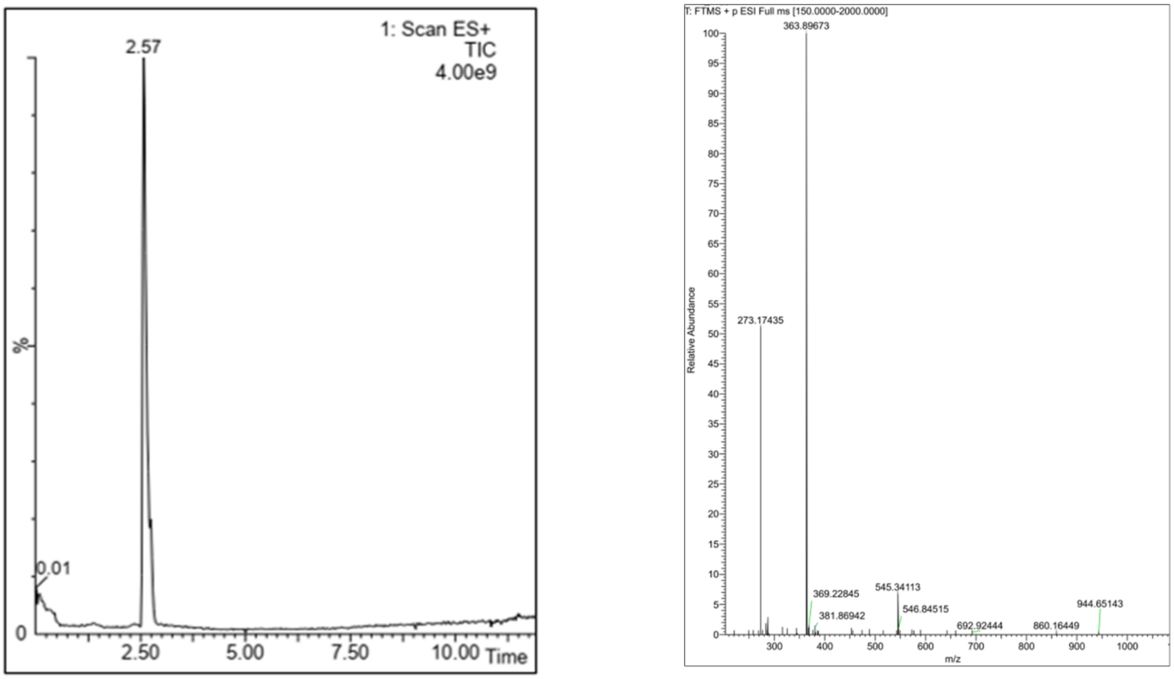

Supplementary Figure S11. TIC and HRMS spectrum of **PMP-4**

- **Compound PMP-5**

Chemical Formula: $C_{59}H_{108}N_{18}O_{16}$

MW = 1325.62 g/mol

m/z [+2]: calculated = 663.9185 found = 633.9163

m/z [+3]: calculated = 442.6136 found = 442.6125

m/z [+4]: calculated = 332.2120 found = 332.2113

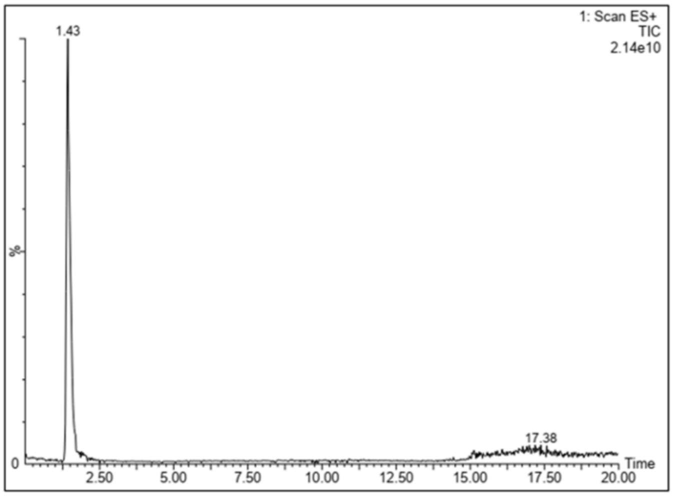

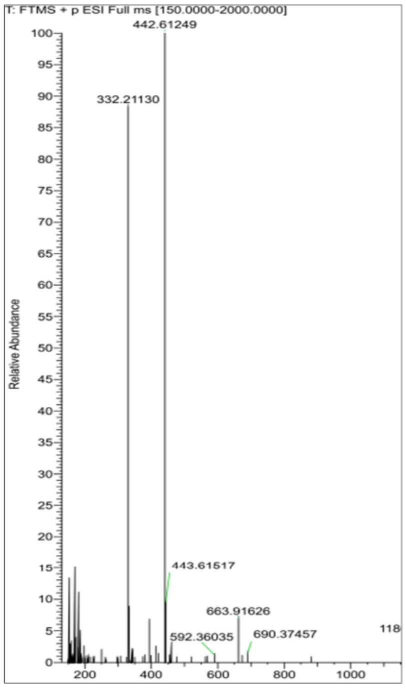

Supplementary Figure S12. TIC and HRMS spectrum of **PMP-5**

3.4 Circular dichroism spectra

Buffer A: acetate buffer (pH 4.75); **Solution B**: NaOH 1M (pH 13)

- **Compound PMP-2**

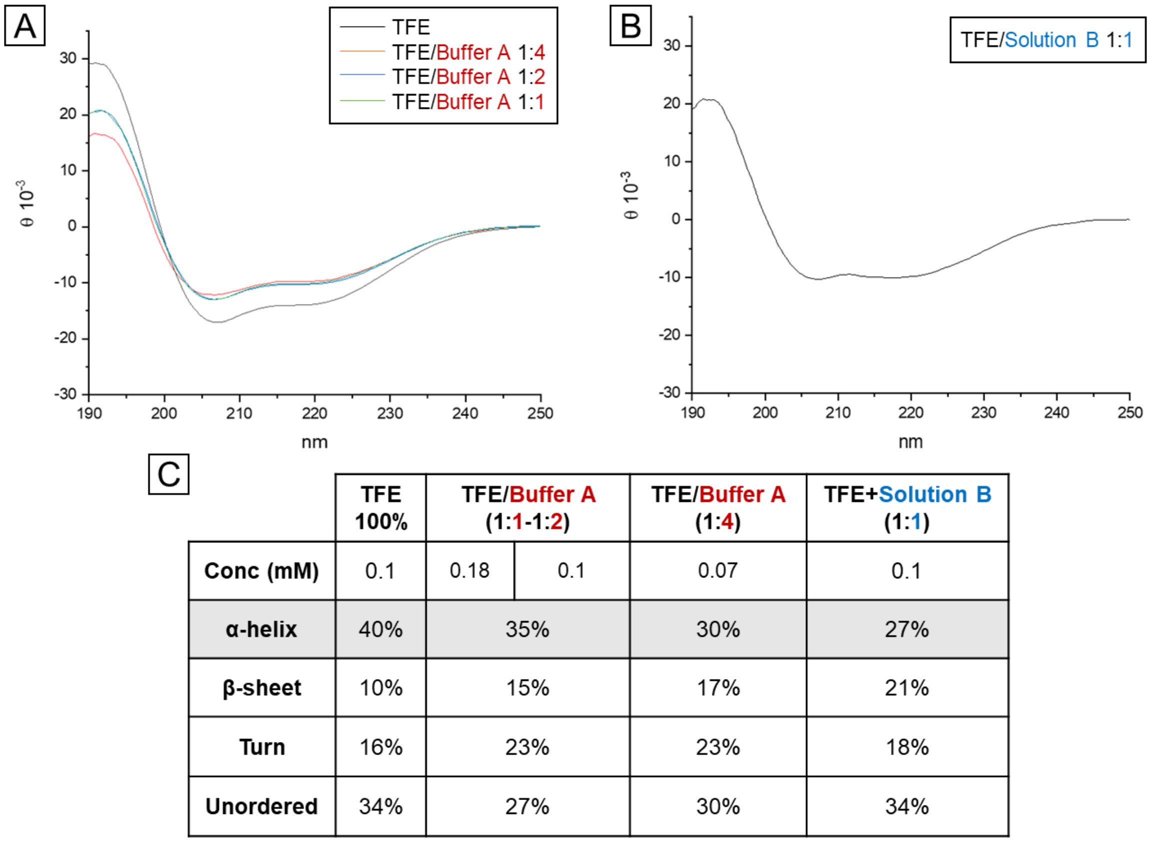

C

	TFE 100%	TFE/Buffer A (1:1-1:2)		TFE/Buffer A (1:4)	TFE+Solution B (1:1)
**Conc (mM)**	0.1	0.18	0.1	0.07	0.1
**α-helix**	40%	35%		30%	27%
**β-sheet**	10%	15%		17%	21%
**Turn**	16%	23%		23%	18%
**Unordered**	34%	27%		30%	34%

Supplementary Figure S13. CD spectra of **PMP-2** in **(A)** pure TFE and mixtures of TFE/acetate buffer 1:1, 1:2, 1:4 and **(B)** TFE/NaOH 1M 1:1. **(C):** Summary table of secondary structure analysis.

- **Compound PMP-3**

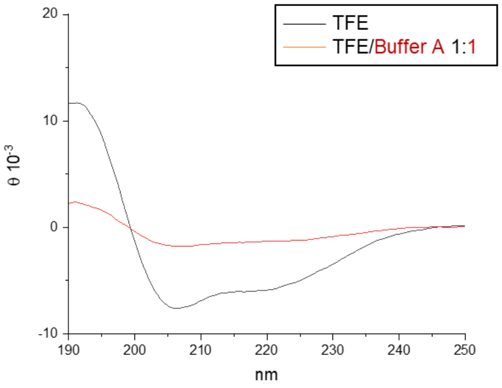

	TFE 100%	TFE/Buffer A 1:1
**Conc (mM)**	0.1	0.18
**α-helix**	19%	7%
**β-sheet**	25%	34%
**Turn**	17%	19%
**Unordered**	39%	40%

Supplementary Figure S14. CD spectra and relative secondary structure analysis of **PMP-3**.

- **Compound PMP-4**

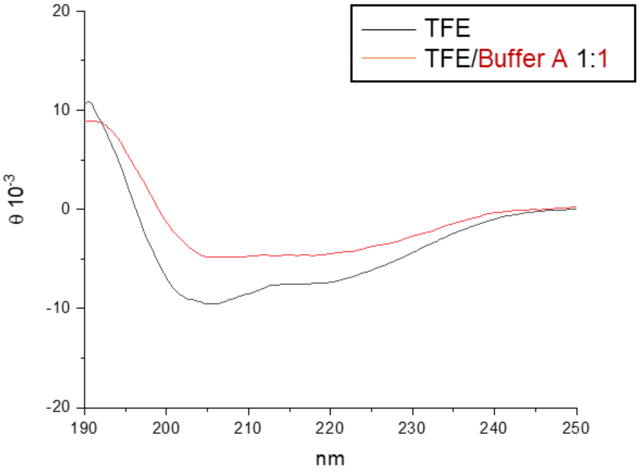

	TFE 100%	TFE+Buffer A 1:1
**Conc (mM)**	0.1	0.1
**α-helix**	18%	12%
**β-sheet**	21%	32%
**Turn**	16%	19%
**Unordered**	45%	37%

Supplementary Figure S15. CD spectra and relative secondary structure analysis of **PMP-4**.

- **Compound PMP-5**

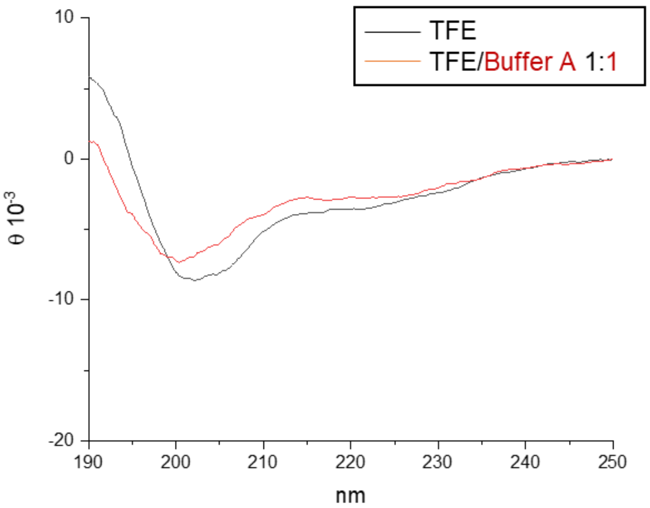

	TFE 100%	TFE+Buffer A 1:1
**Conc (mM)**	0.1	0.1
**α-helix**	12%	8%
**β-sheet**	23%	25%
**Turn**	16%	15%
**Unordered**	49%	52%

Supplementary Figure S16. CD spectra and relative secondary structure analysis of **PMP-5**.

4 Aggregation analysis

4.1 SEM and TEM images

Stock solution 1: **PMP-2** 20 mM in DMSO

Stock solution 2: **PMP-3** 34 mM in DMSO

Stock solution 3: **PMP-4** 46 mM in DMSO

Stock solution 4: **PMP-5** 38 mM in DMSO

- **Compound PMP-2**

SEM images

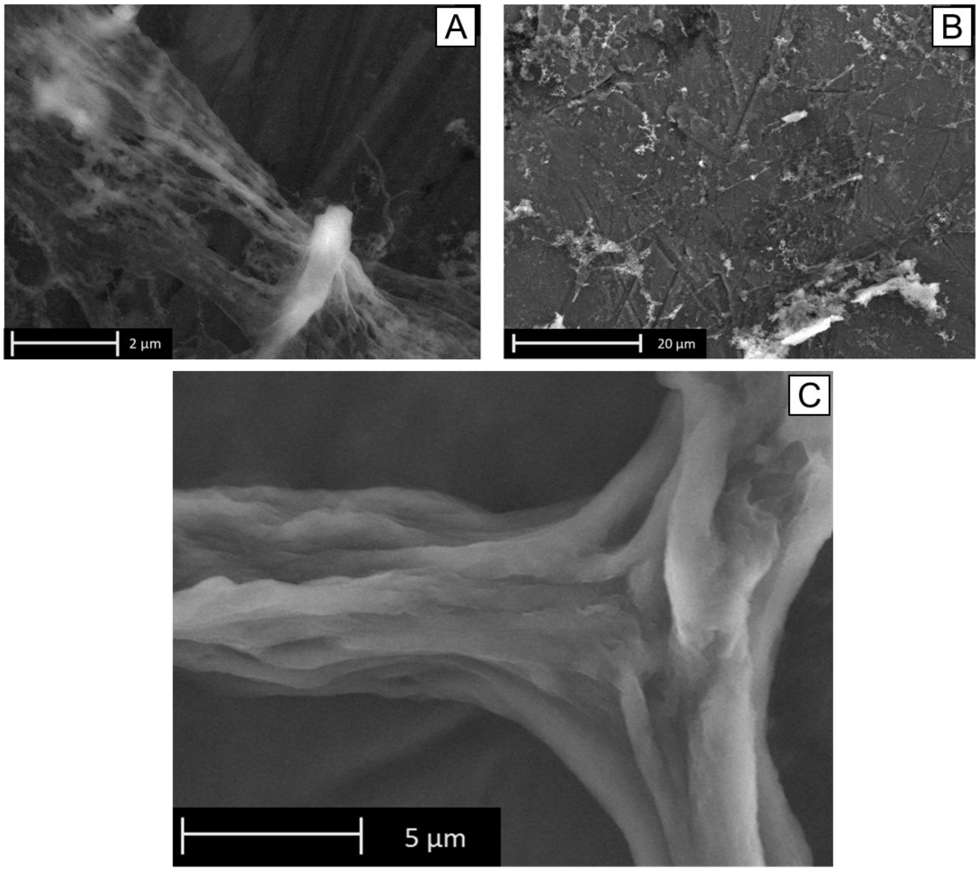

Supplementary Figure S17. SEM images of **PMP-2** after solvent displacement at pH 4 (**A**) and pH 11 (**B**). (**C**) shows a fiber ensemble generating a fiber of larger dimensions at pH 7.

TEM images

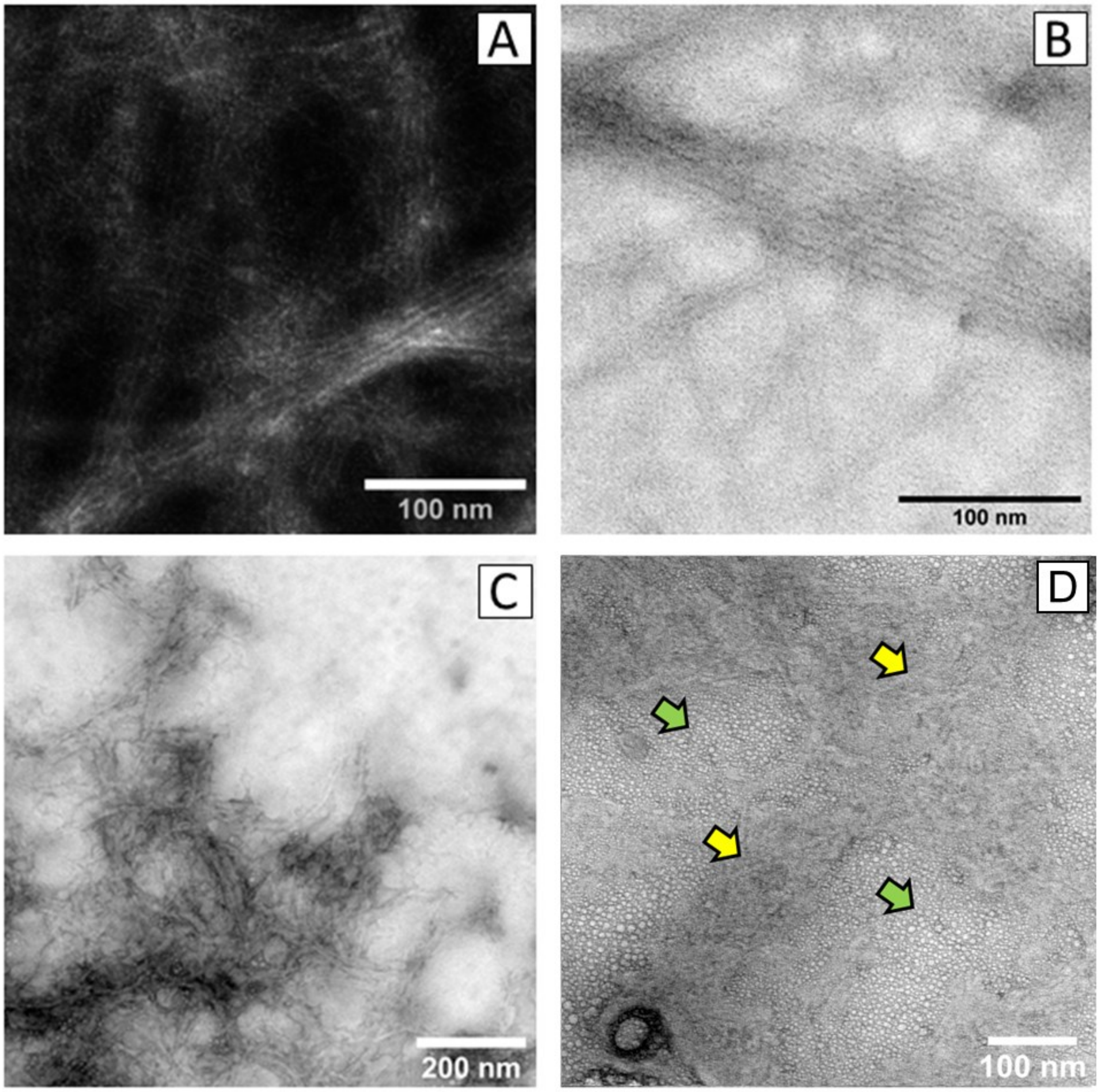

Supplementary Figure S18. TEM/STEM micrographs of **PMP-2** after solvent displacement at pH 4 (**A-B**) highlighting a small ensemble of parallel fibers, pH 7.6 (**C**) and pH 11 (**D**). In (**D**) yellow arrows indicate the presence of small fibrillar aggregates, while green arrows highlight the spherical objects.

- **Compound PMP-3**

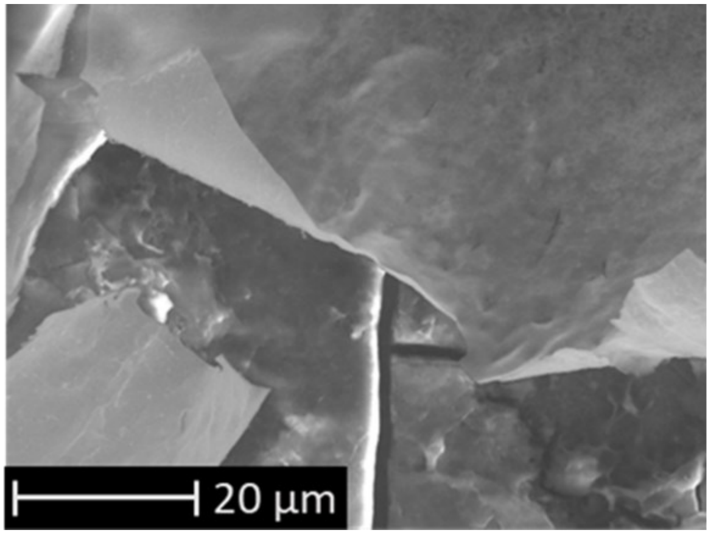

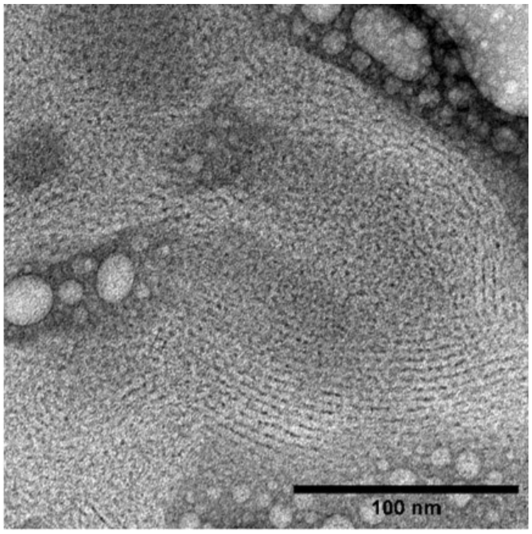

Supplementary Figure S19. SEM (left) and TEM (right) images of **PMP-3** in milliQ water at pH 7.6.

- **Compound PMP-4**

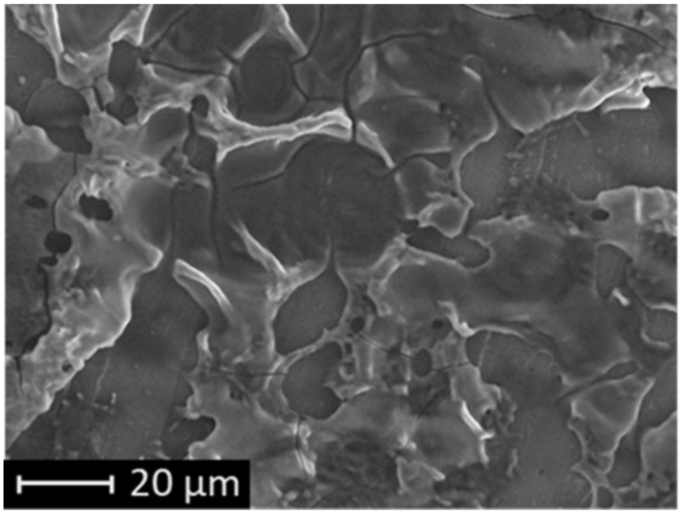

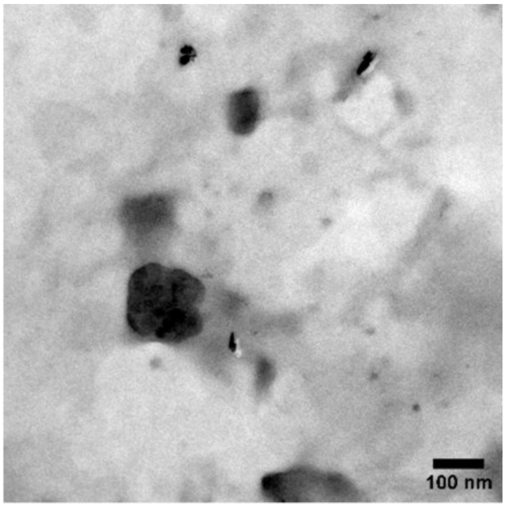

Supplementary Figure S20. SEM (left) and TEM (right) images of **PMP-4 3** in milliQ water at pH 7.6.

- **Compound PMP-5**

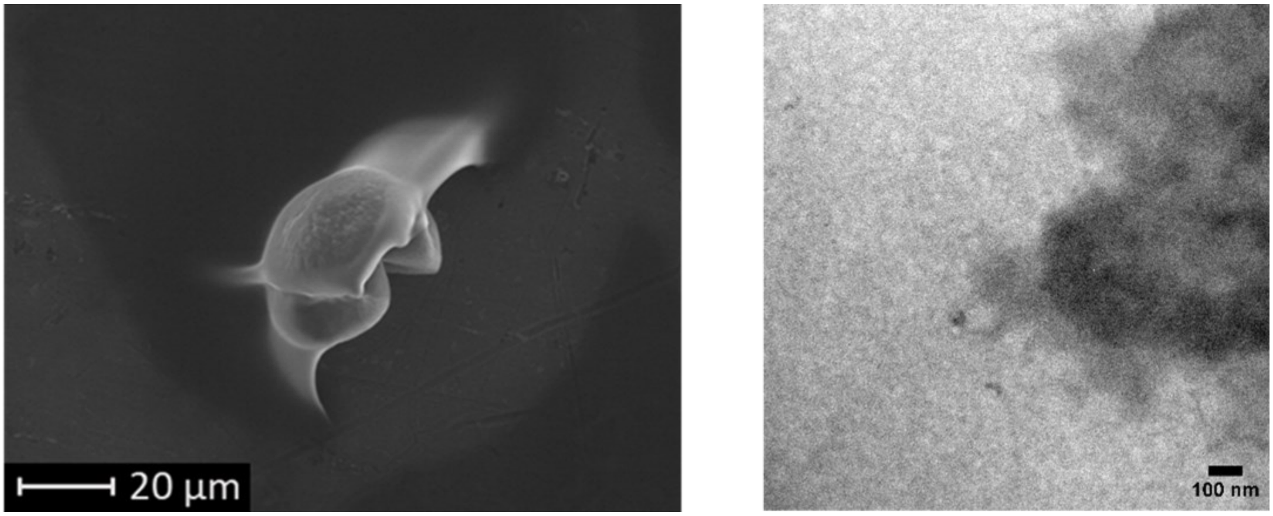

Supplementary Figure S21. SEM (left) and TEM (right) images of **PMP-5** in milliQ water at pH 7.6.

4.2 FT-ATR spectra

All the samples were recorded in solution state, followed by a subtraction of the solvent and the deconvolution process.

- **PMP-2 in solution**

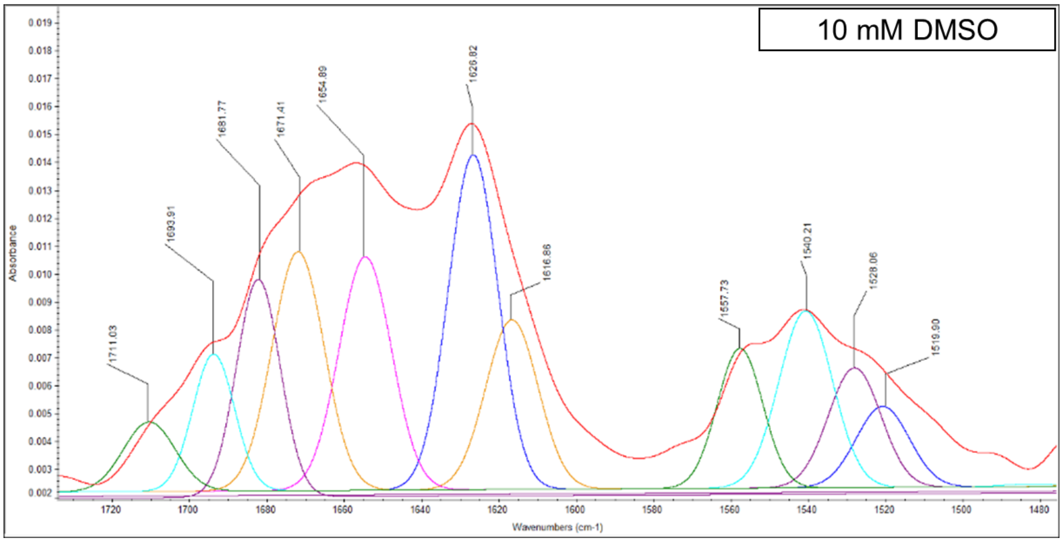

Supplementary Figure S22. ATR spectrum of **PMP-2** DMSO solution with concentration 10 mM.

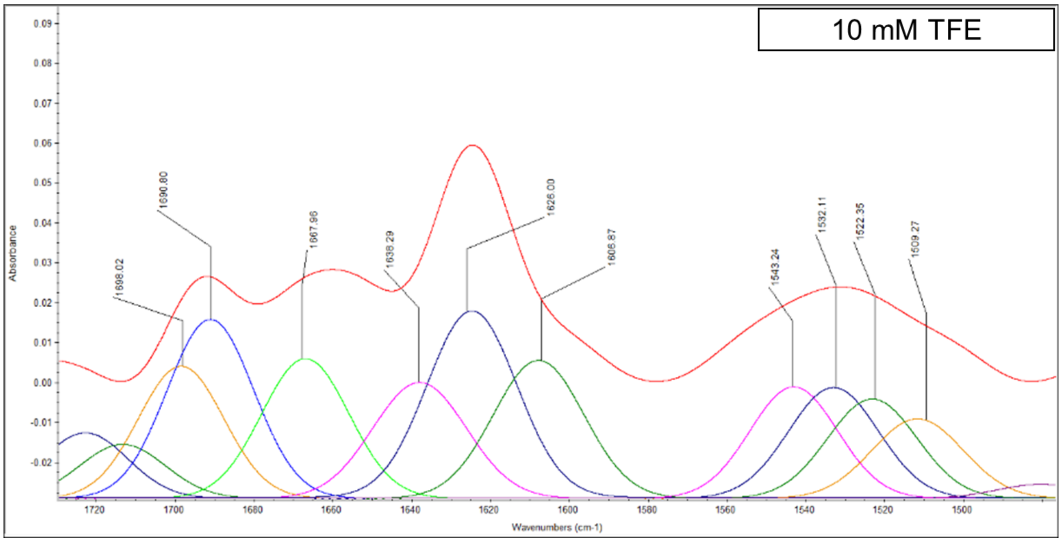

Supplementary Figure S23. ATR spectrum of **PMP-2** TFE solution with concentration 10 mM.

- **<u>PMP-2 hydrogels: 20, 10, 5 and 2 mM</u>**

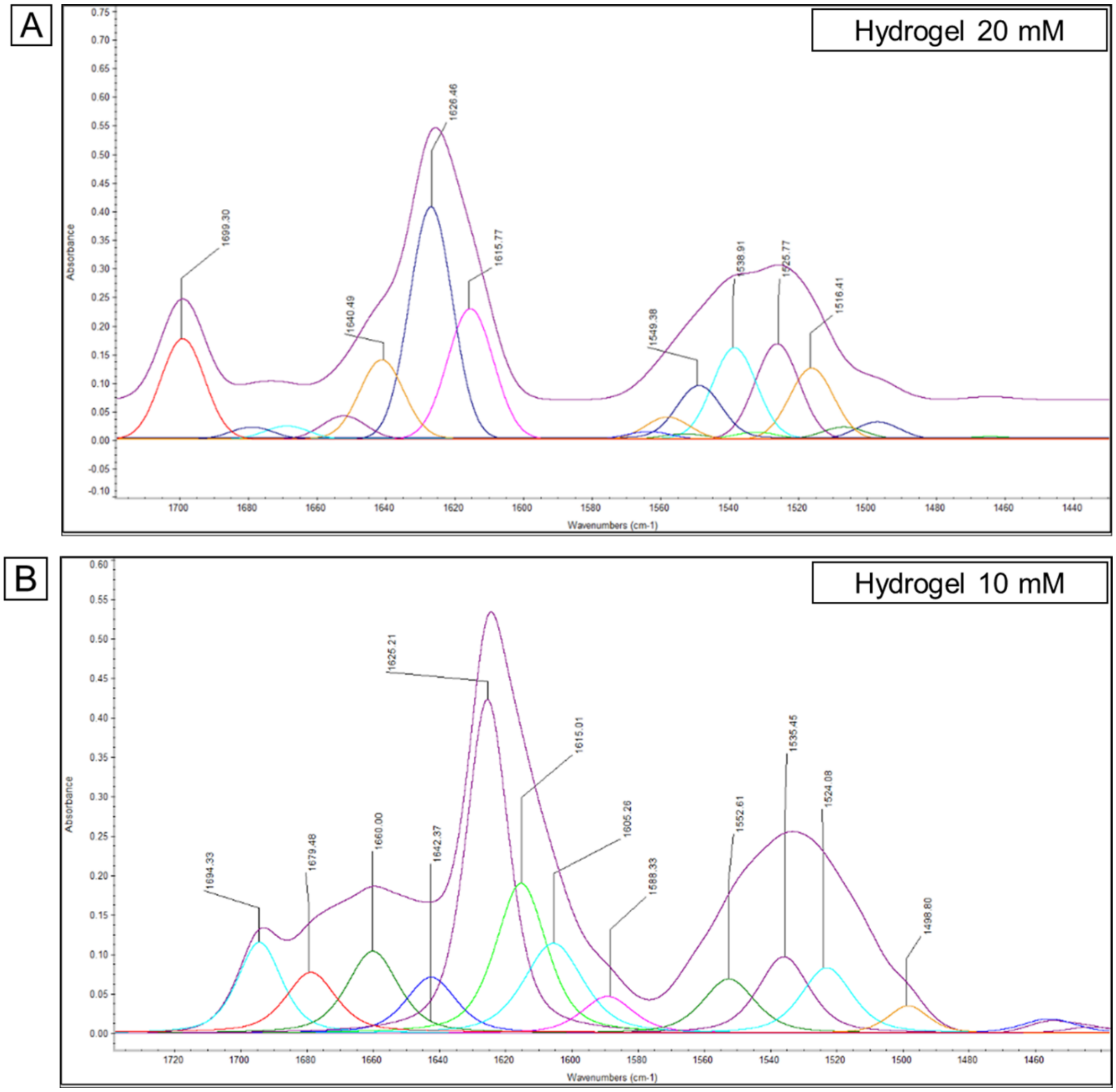

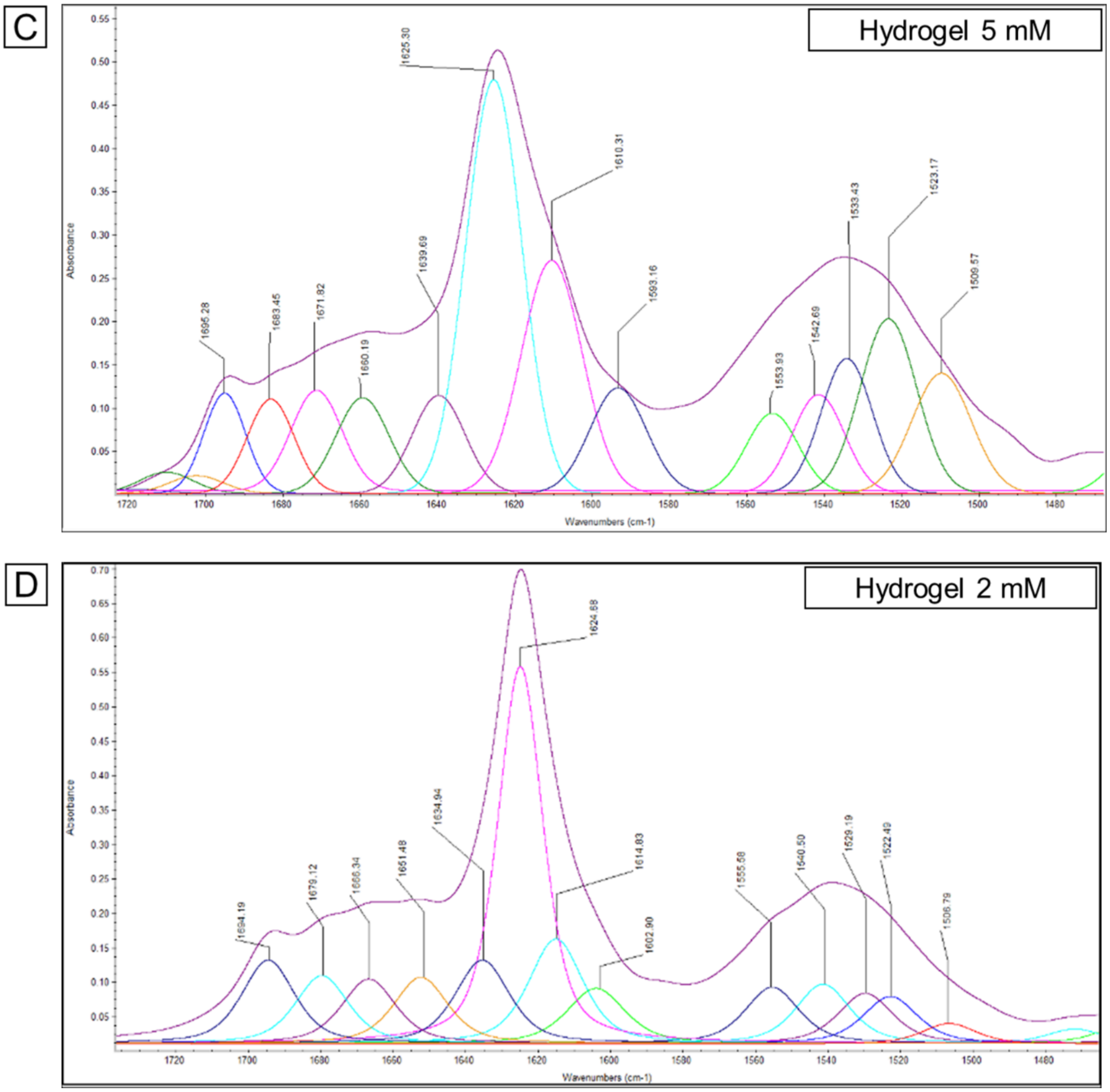

Supplementary Figure S24. Amide I-II region of ATR spectra of **PMP-2** hydrogels at (**A**) 20 mM, (**B**) 10 mM, (**C**) 5 mM and (**D**) 2 mM in milliQ water.

5 Preparation of the PMP-2-based hydrogels

PMP-2 was dissolved in the suitable amount of milliQ water (final concentration of the prepared solutions: 0.1 mM, 1 mM, 2 mM, 5 mM, 10 mM and 20 mM) heating at 60 °C for 3 minutes and sonicating for 1 min. The clear solutions were left cooling at rt. After 24 hours of aging the hydrogel formation was observed at the concentration above 2 mM. The 20 mM hydrogel was kept at room temperature for 10 months, and it showed complete stability.

The GMC (2 mM) was calculated by the vial inversion test.

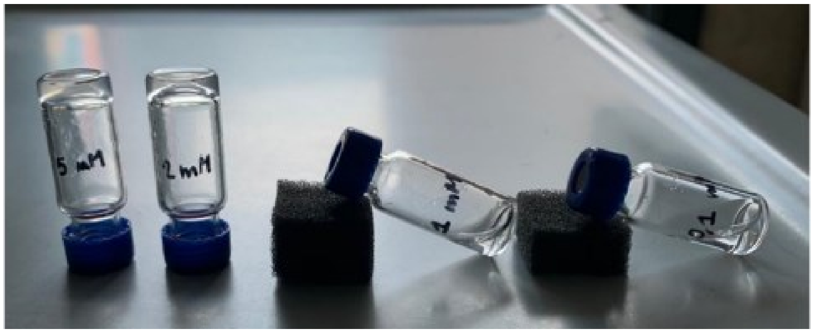

Supplementary Figure S25. Pictures of the **PMP-2** based hydrogels at different concentration in inverted vials. **A.** 20 mM, **B.** 10 mM, **C.** 5 mM and 2 mM, **D.** 1 mM and 0.1 mM in milliQ water.

6 References

Sreerama, N., Woody, R. W. (2000). Estimation of protein secondary structure from circular dichroism spectra: comparison of CONTIN, SELCON, and CDSSTR methods with an expanded reference set. *Anal. Biochem.* 287(2), 252-260. doi: 10.1006/abio.2000.4880

Whitmore, L., & Wallace, B. A. (2008). Protein secondary structure analyses from circular dichroism spectroscopy: methods and reference databases. *Biopolymers.* 89(5), 392-400. doi: 10.1002/bip.20853
